# Readiness to deliver integrated cardiovascular, kidney and metabolic care in primary healthcare: phase II of HEARTS 2.0 in 26 countries in the Americas

**DOI:** 10.1136/bmjgh-2025-021298

**Published:** 2026-01-14

**Authors:** Pedro Ordunez, Andres Rosende, Jeffrey Brettler, Esteban Londono, Patrick Van der Stuyft, Ramon Martinez-Piedra, Libardo Rodriguez, Mariana Lisbeth Rodriguez de la Cerda, Kerry-Ann Renaud-Thomas, Vicente Aleixandre Benites-Zapata, Yadexy Carbay, Maria Clapperton, Miguel Angel Diaz Aguilera, Roxana Salamanca Kacic, Leeann Sills, Salvador Tamayo Muñiz, Hannah Carolina Tavares Domingos, Jerry Toelsie, Yamile Valdes Gonzalez, Natalia Vensentini, Matias Villatoro, Sonia Angell

**Affiliations:** 1 Department of Noncommunicable Diseases and Mental Health, Pan American Health Organization, Washington, District of Columbia, USA; 2 Kaiser Permanente Bernard J Tyson School of Medicine, Pasadena, California, USA; 3 Public Health and Primary Care, Ghent University Faculty of Medicine and Health Sciences, Ghent, Belgium; 4 Ministry of Health of Grenada, Saint George, Grenada; 5 Universidad San Ignacio de Loyola, Lima District, Lima Region, Peru; 6 Pan American Health Organization Venezuela, Caracas, Dto. Capital, Bolivarian Republic of Venezuela; 7 Government of the Republic of Trinidad and Tobago Ministry of Health, Port of Spain, Trinidad and Tobago; 8 National Center for Preventive Programs and Disease Control (CENAPRECE), Ministry of Health of Mexico, Mexico City, Mexico; 9 Ministry of Health and Sport, La Paz, La Paz Department, Plurinational State of Bolivia; 10 Turks and Caicos Islands Ministry of Health, Cockburn Town, Turks Islands, Turks and Caicos Islands; 11 Ministry of Public Health of Cuba, Havana, Cuba; 12 Ministry of Health of Brazil, Brasilia, Brazil; 13 Anton de Kom University of Suriname, Paramaribo, Paramaribo District, Suriname; 14 Government of Argentina Ministry of Health, Buenos Aires, Argentina; 15 Republic of El Salvador Ministry of Health, San Salvador, San Salvador Department, El Salvador; 16 Department of Epidemiology, Johns Hopkins Bloomberg School of Public Health, Baltimore, Maryland, USA; 17 Department of Medicine, Columbia University Medical Center, New York, New York, USA

**Keywords:** hypertension, prevention strategies, health systems, cardiovascular disease

## Abstract

WHO’s Global HEARTS is the largest worldwide effort to improve hypertension control through standardised care. HEARTS in the Americas is its regional adaptation. To address the rising burden of cardiovascular, kidney and metabolic conditions, the initiative launched HEARTS 2.0, aiming to promote integrated care, reduce fragmentation and improve quality, access and health outcomes. In phase I, an expert-led consensus identified 45 evidence-based interventions for inclusion in an expanded Clinical Pathway. This report presents findings from phase II on the readiness of 26 Latin American and Caribbean countries to implement these interventions. We used a cross-sectional design and a structured, self-administered questionnaire completed by national implementation teams. It systematically assessed the availability, feasibility, time required and key barriers for each proposed intervention. While many interventions, especially for risk assessment and non-pharmacological treatments, are considered feasible in many countries, their current availability is limited due to ongoing shortages of diagnostics, medicines and infrastructure. Over the next 3 years, 18 countries are projected to implement >30 of the 45 interventions, four countries aim to implement 20–30 and four expect to implement fewer than 20. While primary health systems in most HEARTS-implementing countries do not yet appear ready to deliver integrated cardiovascular, kidney and metabolic care, the scale-up of HEARTS 2.0 presents a strong opportunity to advance this integration. As health systems worldwide face the challenge of increasing multimorbidity in their patients and fragmented care delivery systems, this assessment offers a practical tool for planning and action.

**Summary box**

- Cardiovascular, kidney and metabolic (CKM) conditions cause over one-third of global deaths and benefit from integrated, person-centred care due to shared risks factors and interconnected pathophysiology.
- HEARTS in the Americas is a regional initiative to improve hypertension management and cardiovascular prevention through standardised care, offering a comprehensive platform that supports a coordinated, primary care-centred approach to reduce fragmentation and enhance quality, access and health outcomes.
- HEARTS 2.0 was launched to expand the existing Clinical Pathway, its main clinical and implementation tool, by incorporating CKM interventions: phase I identified 45 candidate interventions for integration; now, phase II evaluates country readiness for implementing these interventions and phase III will focus on prioritising interventions for scale-up.
- In phase II, 26 Latin American and Caribbean countries completed a cross-sectional assessment of their readiness level to implement the 45 CKM improvement interventions.
- Readiness was evaluated across four dimensions: availability, feasibility, time to scale and barriers.
- Most health systems in HEARTS-implementing countries seem not yet ready to implement integrated CKM care in primary care.
- Pharmacological treatment and service delivery are the weakest components, hindered by high costs, regulatory barriers and cultural issues.

## Introduction

 Cardiovascular, kidney and metabolic (CKM) conditions are the leading cause of death and disability globally.^1^ Combined, they are responsible for an estimated 20–22 million deaths annually, representing about 36%–39% of all global deaths.^2^ These inter-related diseases often coexist and share common risk factors.^3^ Together, they significantly increase the burden of illness on both individuals and health systems.^4^ Addressing this growing syndemic requires integrated, scalable and patient-centred models of healthcare.^5^ Among all health system platforms, primary healthcare (PHC) offers the most strategic and cost-effective entry point for response because of its broad reach, continuity, multidisciplinary orientation and capacity for early intervention.^6^

WHO’s Global HEARTS Initiative was launched in 2016 to strengthen cardiovascular disease (CVD) prevention, primarily by improving hypertension control through standardised care delivery.^7^ In the Americas, this initiative has evolved into a comprehensive regional effort led by the Pan American Health Organisation (PAHO).^8 9^ To date, the HEARTS in the Americas initiative is being implemented in over 7200 PHC centres across 28 Latin American and Caribbean countries (LAC) and territories.^10^

While HEARTS continues to expand its presence in LAC, it is increasingly evident that a focus on hypertension is insufficient. Obesity and diabetes, two major risk factors for cardiovascular and kidney disease, continue to increase in the region, driving CKM rates and related morbidity and mortality. In addition, the increased recognition of CKM syndrome stresses the urgent need for innovative clinical and health services approaches.^3^ CKM syndrome reflects the complex, synergistic interplay among these risk factors, including dyslipidaemia, which collectively impair cardiac, renal and metabolic function. This interconnected dysfunction accelerates the development of CVD and chronic kidney disease (CKD), which, in turn, exacerbates each other in a harmful, self-perpetuating cycle.^11^

Moreover, CKM conditions impose a significant financial strain on health systems. In many LAC countries, where health systems are already under-resourced, strengthening PHC to enable early detection, management and integration of CKM conditions is clinically sound and an effective strategy to reduce long-term system costs and improve equity in access to care.^12 13^

In response, countries participating in HEARTS have increasingly advocated for expanding the HEARTS Clinical Pathway,^14^ its main clinical and implementation tool, to comprehensively address multimorbidity in PHC settings.^15^ In parallel, PAHO’s *Better Care for NCDs Initiative* is emphasising the regional need for integrated service delivery models that align with patient needs and health system realities.^16^

In 2024, HEARTS in the Americas launched HEARTS 2.0, a three-phased strategy to integrate CKM care into HEARTS.^17^ In phase I, an expert panel convened to identify synergistic interventions for hypertension, CVD, diabetes mellitus and CKD that could be integrated into the existing Clinical Pathway. Phase II of HEARTS 2.0 focuses on assessing countries’ readiness to implement the proposed interventions. In phase III, the initiative will determine which interventions will eventually be included in the updated pathway and define strategies for broader adoption. This stepwise approach emphasises active participation from countries and stakeholders, reinforcing the inclusive and evidence-based nature of the HEARTS in the Americas initiative.

In this practice report, we focus on phase II. We describe our design and approach to conducting a LAC assessment that aims to assess healthcare systems’ readiness to incorporate a host of CKM candidate interventions, identified in phase I. We present our findings and discuss their implications for policy and the expansion of the HEARTS programme in LAC. This approach and its findings may serve as a model for assessing and contextualising the integration of CKM interventions into PHC in other regions worldwide.

### Candidate interventions to improve cardio-kidney-metabolic care in primary healthcare

In phase I, an international panel of 59 experts identified a set of 45 evidence-based interventions for hypertension, diabetes, CKD and CVD as candidates for integration into the HEARTS initiative.^17^ The methods have previously been reported^17^ and the list of candidate interventions for integration is provided in table 1.

**Table 1:** Candidate interventions identified in HEARTS 2.0 phase I, categorised by area and recommended action to enhance the HEARTS Clinical Pathway*

Intervention areas	Recommended action	Candidate interventions
Diagnosis	Reinforce	Exclusive use of clinically validated BPMD.
		Improving the clinical environment for accurate BP measurement.
	Include	Expanding community outreach for hypertension screening.
		Set BP diagnostic thresholds for hypertension at ≥140/90 mm Hg in the general population and SBP ≥130 mm Hg for patients at high cardiovascular risk.
Risk assessment	Modify	Set CKD definition: eGFR <60 mL/min and/or uACR index ≥30 mg/g.
		Set BP goals in elderly patients to SBP <130 mm Hg.
		Define a CVD risk approach for young adults (aged 18–40 years).
	Include	CKD screening for high-risk individuals using uACR and eGFR.
		Assessment of HTN-mediated organ damage with ECG in patients with high CVD risk.
		Screening for dyslipidaemia and diabetes in patients with HTN and obesity.
		Opportunistic screening for atrial fibrillation in patients with high CVD risk of any age and those aged ≥65 years.
		Closely monitor individuals with a history of hypertension during pregnancy.
		Add a warning: avoid treatment with short-acting and parenteral agents in individuals with severe, asymptomatic, uncontrolled hypertension.
Non-pharmacological treatment	Include	Promote low-sodium/potassium-enriched salt.
		Prescribe isometric exercise.
		Warning against smoking cannabis.
		Warning against electronic cigarette use/vaping.
		Avoid a sedentary lifestyle.
		Prescribe exercise.
Pharmacological treatment	Reinforce	SPC antihypertensive medicines.
	Modify	Add the third drug, at half maximum dose, in the second step of the treatment protocol instead of increasing the first two drugs to maximum doses.
		Maximum statin doses in secondary prevention (atorvastatin 80 mg or rosuvastatin 40 mg).
		High statin doses in primary prevention (atorvastatin 40 mg or rosuvastatin 20 mg).
		Use of polypills (antihypertensives plus statins with or without aspirin) for primary and secondary prevention of CVD.
		Intensify antihypertensive medication at intervals of 2 weeks instead of 4 weeks.
		Warning on assessing childbearing potential before treatment initiation.
	Include	Triple SPC for those patients who do not reach BP control using double SPC.
		Spironolactone in patients on three drugs at maximum doses and lack of HTN control.
		Treatment for tobacco cessation (bupropion, varenicline, nicotine substitutes).
		SGLT2i in patients with CKD.
		SGLT2i in patients with heart failure.
		SGLT2i in patients with diabetes and established CVD.
Continuity of care	Modify	Intensive BP (SBP <130 mm Hg) goals restricted to patients aged <80 years.
	Include	Home BP treatment monitoring.
		Telemedicine/mHealth apps to monitor recommendation adherence and to reduce loss to follow-up.
		Lipid targets in patients with high CVD risk.
		An established target time to achieve BP control.
		Warning to avoid statin discontinuation once the control target has been reached.
Delivery system	Include	Medication intensification by NPHWs following a protocol.
		HTN screening and CVD risk stratification by NPHWs.
		Healthy lifestyle counselling and support for medication adherence by NPHWs.
Immunisation	Modify	Influenza vaccination to all patients with HTN regardless of CVD risk level.
		Pneumococcus vaccination should exclude patients aged <65 years in primary prevention.
System for monitoring	Include	Importance of registering clinical variables.
		Relevance of having a strategy of performance evaluation with feedback.

* Adapted from Rosende *et al*.^17^

BP, blood pressure; BPMD, blood pressure measuring devices; CKD, chronic kidney disease; CVD, cardiovascular disease; eGFR, estimated glomerular filtration rate; HTN, hypertension; NPHWs, non-physician health workers; SBP, systolic blood pressure; SGLT2i, sodium-glucose co-tranporter-2 inhibitors; SPC, single pill combination; uACR, urine albumin-to-creatinine ratio.

### HEARTS 2.0 phase II: assessing readiness to implement cardiovascular, kidney and metabolic care interventions in primary healthcare

#### Survey development and design

Following phase I, the dialogue expanded to include national implementation teams and local stakeholders in HEARTS-participating countries. In late 2024, a cross-sectional readiness assessment design using a structured, self-administered questionnaire (see ‘HEARTS 2.0—readiness assessment tool’ in online supplemental material) was conducted to assess the programme integration of CKM care into PHC, focused on the 45 candidate interventions identified in phase I (table 1).

Each country followed a collective, participatory process led by the national HEARTS coordination team appointed by the Ministry of Health. Countries were encouraged to involve a broad range of technical experts, procurement and health technology officials, PHC officers and Ministry representatives. A single questionnaire was completed and submitted per country, reflecting the consensus reached after internal deliberations among relevant entities within the Ministry of Health. In May 2025, all countries revisited and updated their responses to reflect any changes since the initial evaluation submission (see ‘HEARTS 2.0—readiness assessment results’ in online supplemental material).

In the survey, readiness was evaluated across four dimensions: (a) reported availability of candidate interventions, (b) perceived feasibility of implementation of candidate interventions, (c) expected time to implement and (d) discerned barriers.

Each candidate intervention was appraised on a 9-point scale (1=lowest, 9=highest) for both availability and implementation feasibility. Availability refers to the presence of essential inputs and resources needed to operationalise these interventions, reflecting what is currently in place and accessible within the health system. Implementation feasibility measures how effectively these interventions can be executed in practice, considering existing infrastructure, service delivery workflows, institutional capacity and policy frameworks.

Moreover, countries were asked to specify whether each intervention was already considered a standard practice, defined as one widely accepted and routinely used by health professionals as the recommended care method. For interventions not yet recognised as standard practice, countries estimated the time required to fully integrate them into routine PHC delivery, categorised as <1 year, 1–2 years or >3 years.

Finally, countries were asked to identify the main barrier that limits the implementation of each intervention. Barriers were sorted into three groups: (a) cost-related barriers, which include financial issues like the price of medications, equipment, training, staffing or infrastructure; (b) regulatory barriers, such as laws or policies that limit implementation, including restrictions on scope of practice or required treatment approvals and (c) cultural barriers, referring to social beliefs, behaviours or attitudes that can block adoption, like mistrust in health services or providers, resistance to change or lack of cultural relevance.

#### Survey evaluation

Data were analysed using a structured approach based on descriptive statistics. Frequency distributions were used to assess the spread of responses across countries for each of the four assessment dimensions. For reported availability and perceived feasibility of implementation of candidate interventions, response scores were categorised as: low (1–3), medium (4–6) or high (7–9). Time to implementation was summarised using response proportions in each predefined category (current standard of practice, <3 years, >3 years), while barriers were categorised and tallied by type (economic, regulatory or cultural). Figures were developed to identify cross-country patterns and highlight gaps and opportunities.

#### Survey findings

**Figure 1:**
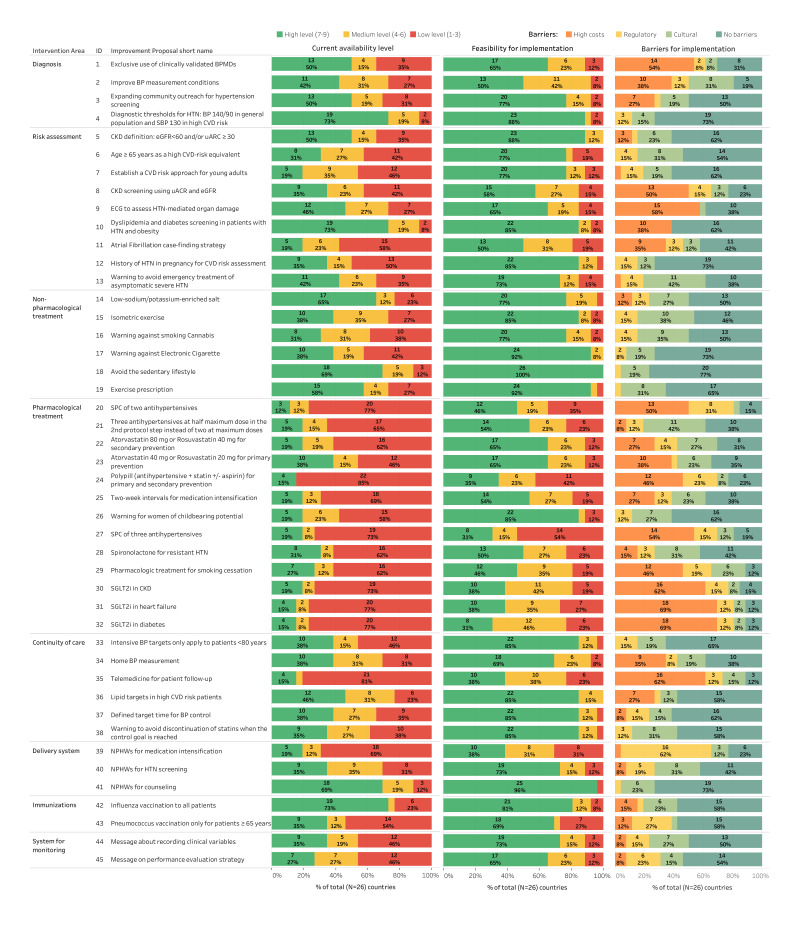
Levels of current availability and implementation feasibility for each intervention, grouped by intervention area and the main implementation barriers. BP, blood pressure; BPMD, blood pressure measuring devices; CKD, chronic kidney disease; CVD, cardiovascular disease; eGFR, estimated glomerular filtration rate; HTN, hypertension; NPHWs, non-physician health workers; SBP, systolic blood pressure; SGLT2i, sodium-glucose co-transporter-2 inhibitors; SPC, single pill combination; uACR, urine albumin-to-creatinine ratio.

Of the 33 LAC countries invited, 26 completed the assessment. Figure 1 presents an overview of the availability, implementation feasibility and main barriers of the candidate interventions across participating countries. Each area is colour-coded based on the proportion of countries reporting high, medium or low levels of availability and implementation feasibility (‘online supplemental figure 1).

The intervention area of diagnosis demonstrated one of the relatively highest levels of both availability and implementation feasibility. However, cost remains a significant barrier to the procurement of clinically validated automatic blood pressure measurement devices and the necessary facility infrastructure for accurate blood pressure measurement.

The risk assessment area shows a mixed pattern. There are significant gaps in availability, along with a promising perceived feasibility of implementation. While several countries have implemented key interventions such as screening for dyslipidaemia, diabetes and CKD markers, others fall behind, especially in routinely detecting atrial fibrillation and performing combined CKD screening with urine albumin-to-creatinine ratio (uACR) and estimated glomerular filtration rate. Barriers in this area include cultural obstacles and high costs.

The non-pharmacological treatment area has substantial availability gaps in key interventions. Notably, about 40% of countries have yet to adopt public health warnings regarding electronic cigarette use/vaping. Despite cultural barriers, most countries view this domain as highly feasible for scale-up in the near term.

Pharmacological treatment is the area with the least availability of resources for interventions identified in HEARTS 2.0, phase I. Access to essential medications, including single-pill combinations for hypertension, polypills, sodium-glucose cotransporter-2 inhibitors (SGLT2i) and high-intensity statins, is limited. Cost is the primary barrier, leading to cautious projections for expansion in this area over the coming years.

The continuity of care area reveals significant gaps, for instance, in the availability of telemedicine and home blood pressure monitoring. While other follow-up interventions are considered more feasible, financial constraints continue to hinder broader implementation.

The delivery system area, which emphasises the role of non-physician health workers (NPHWs) in hypertension screening and treatment, indicates poor availability. Among its interventions, treatment intensification by NPHWs was deemed the least feasible, primarily due to regulatory restrictions. In contrast, hypertension screening by NPHWs received a more favourable rating, although it still faced cultural barriers regarding task-sharing.

The Monitoring Systems area remains largely underdeveloped. Few countries have established mechanisms for clinical monitoring, although there is moderate expectation for future development.

Regarding immunisations, influenza vaccination is widely available for all patients with hypertension. Conversely, restricting pneumococcal vaccination to individuals over 65 years, thereby avoiding its use in younger patients with high CVD risk without end-organ damage, is perceived as feasible but faces regulatory barriers.

Cost was the most frequently cited top one barrier, particularly affecting the diagnosis and pharmacological treatment areas, followed by regulatory and cultural challenges, mainly in the delivery system and non-pharmacological treatment area, respectively.

#### Timeline for the rollout of interventions identified in HEARTS 2.0, phase I

**Figure 2:**
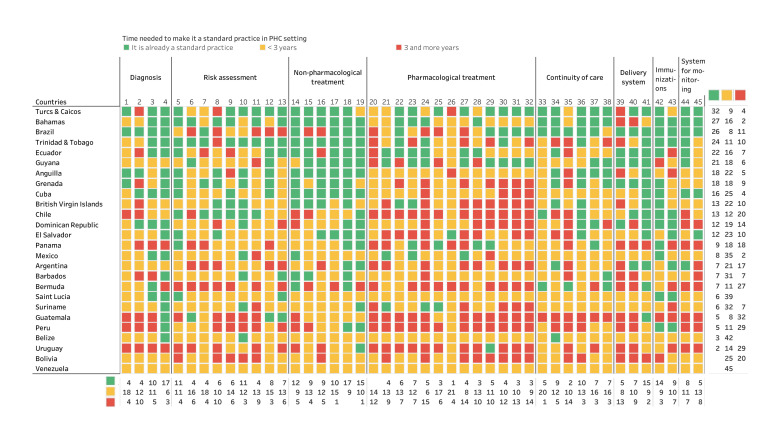
Expected timeline for implementing HEARTS 2.0 candidate interventions within the next 3 years by the country and intervention areas.

Figure 2 shifts the focus to the expected timeline for implementing interventions identified in phase I. The expected timeline varies considerably across countries. Eighteen countries (69.2%) have already implemented or are on track to implementing >30 interventions within 3 years, indicating high readiness. Four countries (15.4%) expect to implement between 20 and 30 interventions within 3 years, reflecting moderate readiness. The remaining four countries (15.4%) anticipate implementing fewer than 20 interventions within this time, suggesting more limited capacity.

These data suggest that countries’ capacity to implement these interventions is highly contextual, depending on economic resources and on the political and economic environment at the time the information was collected. Other important factors include political will, the organisation and strength of the health system, regulatory frameworks, workforce capacity and local cultural and social norms. Together, these contextual elements can strongly influence the effectiveness of implementation and the scaling-up of interventions within health services.

Each row represents a country, and each column refers to a specific candidate intervention, identified with a number from 1 to 45 and grouped into intervention areas. Colour-coded cells reflect the three categories of adoption timelines for each country and intervention: green for standard practice, yellow for feasible within 3 years and red for expected implementation beyond 3 years. On the right, the figure shows the number of interventions by adoption timeframe category for each country. At the bottom, the figure shows the number of countries by adoption timeframe category for each intervention.

### Limitations and strengths

As HEARTS leadership and participating countries consider strategies for implementing these findings into HEARTS programming in phase III, several limitations and strengths must be kept in mind. First, the evaluation is based on self-reported data, which may introduce bias. The accuracy of responses depends on the knowledge and judgement of the participants. Availability of essential inputs and feasibility of implementation were assessed based on perceptions rather than objective metrics, and for practical reasons, current coverage across health facilities or the proportion of the population reached by each intervention was not quantified. Similarly, estimates of implementation timelines were inherently subjective.

Second, the evaluation primarily focuses on public health services provided by Ministries of Health and may not fully capture the role of private or other providers, which could limit its comprehensiveness. However, in LAC, Ministries of Health serve most of the population, especially vulnerable groups, making this focus highly relevant for public health planning.

Third, as a cross-sectional assessment, it offers a snapshot in time and cannot track changes in readiness or intervention impact over time. Fourth, variation in data quality and completeness across countries may affect the consistency of findings.

While 26 of the 33 countries that committed to implementing HEARTS participated in this assessment, broader engagement could have provided a more comprehensive regional overview. Additionally, the lack of global or regional benchmarks for integrated CKM care limits the ability to objectively compare progress across countries.

Despite these limitations, this assessment provides a valuable baseline to inform strategic planning, technical cooperation and regional coordination within the HEARTS in the Americas initiative. It identifies key system gaps and supports a phased, evidence-informed approach to integrating CKM care into PHC.

### Policy and programme implications

Despite the rising burden of CKM conditions over the past three decades in LAC,^18^ the health systems of most HEARTS-implementing countries still seem not well-equipped to implement the interventions required to integrate CKM care into PHC. Advancing at the pace demanded by this major public health challenge will require strategic policies and substantial resource commitments.

This assessment reveals substantial variability in countries’ readiness to address this major challenge, likely reflecting differences in infrastructure, workforce capacity, financing and governance, as documented in prior studies.^19^ However, this variability cannot be explained by financial resources alone. Some countries with higher gross domestic product than many of their peers have lagged in integrating CKM care. This also might reflect an incomplete understanding of the shared pathophysiology of CKM conditions and highlights the need to overcome entrenched barriers.^20^ Health systems across the region remain anchored in vertical, disease-specific delivery models poorly suited to managing multimorbidity.^21 22^ Moreover, clinical guidelines are often developed in silos and lack the comprehensiveness required for integrated implementation at the PHC level, reinforcing fragmentation and weakening care delivery.^23^ Advancing equitable progress will require addressing deeply structural, strategic and governance shortcomings across health systems.

Against this backdrop, three strategic patterns emerge from the assessment. First, interventions related to diagnostic and risk assessment seem to provide practical entry points for expanded efforts, as they reported the highest levels of availability and feasibility. However, affordability remains a concern, especially regarding the cost of automatic clinically validated blood pressure measurement devices, a key diagnostic tool.^24^

Second, pharmacological treatment remains the weakest area across countries and represents a major bottleneck for scale-up. Access to essential medicines, such as single-pill combination antihypertensives, SGLT2i and statins, is limited due to high prices, procurement inefficiencies and outdated national formularies.^25 26^ Since these barriers are not primarily patent-related and effective therapies already exist, a bold pharmaceutical policy is needed to prioritise price reduction and ensure availability. This includes standardising treatment regimens, strengthening regional manufacturing, improving supply chains and promoting transparent procurement mechanisms.^27^ Expanding the use of PAHO’s Strategic Fund is one effective strategy, offering pooled procurement and access to quality-assured products at competitive prices for LAC countries.^26^ Policies that spread patient management tasks across the care delivery team are also essential to allow timely treatment initiation and dose adjustment by nurses and pharmacists within PHC.^28 29^ Unfortunately, this is hampered by regulatory barriers.

Third, service delivery and monitoring emerge as structural weaknesses. Fragmented service delivery, underutilisation of NPHWs and limited continuity of care continue to undermine integration.^28^ In addition, most countries lack effective monitoring systems, limiting their ability to track progress and make timely course corrections.^30^ These gaps underscore the need for investments in digital infrastructure, strengthened governance and reimagined care models that support team-based, longitudinal CKM management.

The findings from this regional assessment underscore the pressing need to evolve the HEARTS Initiative to go beyond a primary focus on hypertension into a more comprehensive 2.0 framework that systematically integrates the clinical management of CKM disorders.^17^ One of the most compelling insights from the widespread adoption of the HEARTS initiative is that progress is achievable, even in resource-limited settings, when integration is guided by strategic, structured, scalable approach models.^31 32^

Translating this vision into practice requires acknowledging the substantial heterogeneity in the LAC country’s socio-economic development, health system set-up and readiness. A one-size-fits-all implementation strategy is neither practical nor equitable. While some countries are ready to scale integrated interventions, others still face foundational gaps in the workforce, infrastructure, medicines and governance. Bridging these gaps will require differentiated support strategies that are tailored to each country’s stage of readiness and specific system constraints. This does not mean lowering quality standards but rather establishing institutional mechanisms to ensure the best possible treatment reaches everyone, leaving no one behind.

## Conclusions

Most health systems in LAC countries implementing HEARTS seem not yet ready to implement the set of candidate interventions identified by HEARTS 2.0, phase I, to deliver integrated CKM care in the PHC settings. This assessment underscores the scale of the challenge, the strength of the country’s responses and willingness and the strategic opportunities ahead. Rather than emphasising country-specific outcomes or isolated actions, the analysis highlights system-level patterns, structural weaknesses and common barriers that can inform a coordinated regional response.

To translate readiness into results, countries should adopt bold, context-adapted actions that realign financing, governance and service delivery with the complex realities of CKM multimorbidity. Differentiated implementation strategies, tailored to each country’s system capacity, will be key to ensuring sustainable, high-quality and equitable integrated care.

The scale-up of HEARTS V.2.0 presents a strong opportunity to advance the delivery of high-quality, integrated CKM care through a primary care-based model that reduces fragmentation and improves quality, access and health outcomes. As health systems around the world grapple with rising multimorbidity in their patients and a fragmented care delivery system, this assessment provides a critical strategic framework for policymakers, implementers, stakeholders and funders committed to transforming PHC as the cornerstone of NCD control, both in the Americas and globally.

## Supplementary material

10.1136/bmjgh-2025-021298 online supplemental file 1

10.1136/bmjgh-2025-021298 online supplemental file 2

10.1136/bmjgh-2025-021298 online supplemental file 3

## Data Availability

All data relevant to the study are included in the article or uploaded as supplementary information.
