# Supplementary figures and images for: Readiness to deliver integrated cardiovascular, kidney and metabolic care in primary healthcare: phase II of HEARTS 2.0 in 26 countries in the Americas
Source: BMJ Glob Health. 2026 Jan 14;11(1):e021298. doi: 10.1136/bmjgh-2025-021298 (PMC12815231; doi:10.1136/bmjgh-2025-021298)

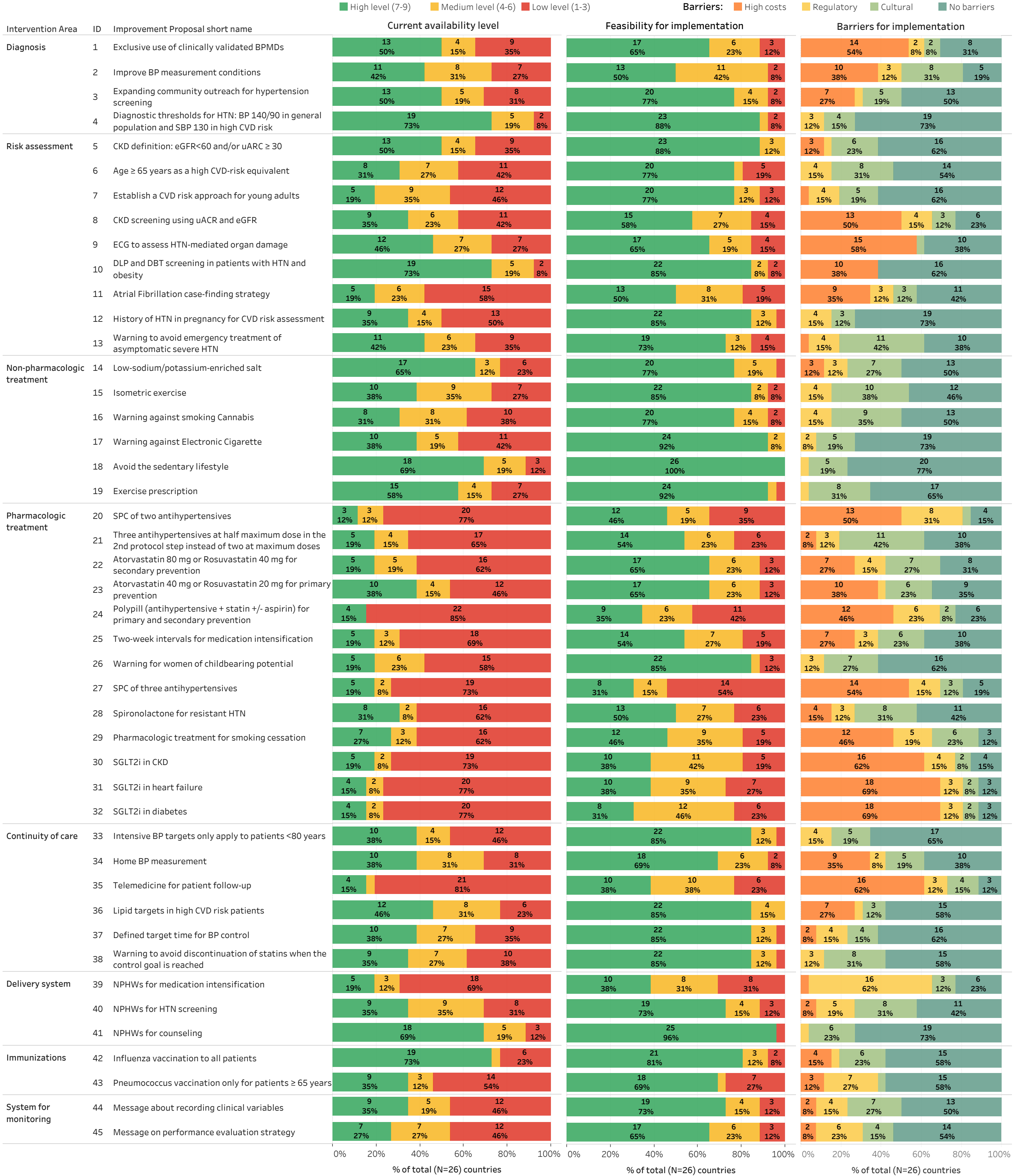

Supplement: online supplemental file 1 [file bmjgh-11-1-s001.pdf]
