# Supplementary material for: Readiness to deliver integrated cardiovascular, kidney and metabolic care in primary healthcare: phase II of HEARTS 2.0 in 26 countries in the Americas
Source: BMJ Glob Health. 2026 Jan 14;11(1):e021298. doi: 10.1136/bmjgh-2025-021298 (PMC12815231; doi:10.1136/bmjgh-2025-021298)
Supplement: online supplemental file 2 [file bmjgh-11-1-s002.pdf]

ID	Intervention Area	Action	Intervention	Countries	Current level of availability in PHC settings	Perceived level of feasibility to be implemented in PHC settings	Time needed to make it a standard practice in PHC settings	Main barrier to implement it in your country
1	Diagnosis	Reinforce	Exclusive use of clinically validated Blood Pressure Measuring Devices to accurate BP measurement	Anguilla	9	9	It is already a standard practice	No barriers
				Argentina	3	9	1 - 2 years	Cultural barriers
				Bahamas	8	9	< 1 year	No barriers
				Barbados	7	9	1 - 2 years	High costs
				Belize	3	8	1 - 2 years	High costs
				Bermuda	7	9	1 - 2 years	Cultural barriers
				Bolivia	3	6	1 - 2 years	Regulatory barriers
				Brazil	9	9	It is already a standard practice	No barriers
				BVI	8	8	< 1 year	High costs
				Chile	6	6	> 3 years	High costs
				Cuba	5	8	< 1 year	High costs
				Dominican Republic	5	7	1 - 2 years	No barriers
				Ecuador	8	8	< 1 year	No barriers
				El Salvador	8	8	< 1 year	High costs
				Grenada	8	9	It is already a standard practice	No barriers
				Guatemala	2	5	> 3 years	High costs
				Guyana	2	1	1 - 2 years	High costs
				Mexico	5	3	1 - 2 years	High costs
				Panama	1	3	1 - 2 years	High costs
				Peru	1	7	> 3 years	High costs
				Saint Lucia	9	9	< 1 year	Regulatory barriers
				Suriname	1	5	1 - 2 years	High costs
				Trinidad & Tobago	7	4	1 - 2 years	High costs
				Turcs & Caicos	9	9	It is already a standard practice	No barriers

				Uruguay	1	6	> 3 years	No barriers
				Venezuela	7	9	< 1 year	High costs
2	**Diagnosis**	**Reinforce**	**Recommendations to improve BP measurement (unobserved, quiet room, etc).**	Anguilla	7	7	It is already a standard practice	Regulatory barriers
				Argentina	5	8	1 - 2 years	High costs
				Bahamas	7	6	1 - 2 years	Regulatory barriers
				Barbados	3	5	> 3 years	Cultural barriers
				Belize	8	8	1 - 2 years	Cultural barriers
				Bermuda	2	7	1 - 2 years	Cultural barriers
				Bolivia	2	5	1 - 2 years	Cultural barriers
				Brazil	9	9	It is already a standard practice	No barriers
				BVI	1	2	> 3 years	High costs
				Chile	9	5	> 3 years	High costs
				Cuba	7	8	It is already a standard practice	No barriers
				Dominican Republic	6	9	It is already a standard practice	No barriers
				Ecuador	4	4	> 3 years	High costs
				El Salvador	8	9	< 1 year	Cultural barriers
				Grenada	4	5	> 3 years	High costs
				Guatemala	2	6	> 3 years	High costs
				Guyana	5	5	1 - 2 years	Regulatory barriers
				Mexico	9	9	< 1 year	No barriers
				Panama	4	3	> 3 years	High costs
				Peru	1	5	> 3 years	High costs
				Saint Lucia	6	8	1 - 2 years	Cultural barriers
				Suriname	4	9	< 1 year	Cultural barriers
				Trinidad & Tobago	7	6	< 1 year	No barriers
				Turcs & Caicos	7	7	> 3 years	High costs
				Uruguay	3	5	> 3 years	High costs

				Venezuela	9	7	< 1 year	Cultural barriers
3	Diagnosis	Include	Expand HTN screening in the community served by each PHC (community engagement).	Anguilla	5	6	1 - 2 years	No barriers
				Argentina	6	8	1 - 2 years	Cultural barriers
				Bahamas	9	9	It is already a standard practice	No barriers
				Barbados	2	5	> 3 years	High costs
				Belize	6	9	< 1 year	Regulatory barriers
				Bermuda	9	9	It is already a standard practice	No barriers
				Bolivia	3	9	1 - 2 years	No barriers
				Brazil	9	9	It is already a standard practice	No barriers
				BVI	5	8	1 - 2 years	High costs
				Chile	2	2	1 - 2 years	High costs
				Cuba	9	9	It is already a standard practice	No barriers
				Dominican Republic	7	9	It is already a standard practice	No barriers
				Ecuador	8	8	It is already a standard practice	Cultural barriers
				El Salvador	8	7	1 - 2 years	High costs
				Grenada	7	9	< 1 year	No barriers
				Guatemala	2	8	> 3 years	High costs
				Guyana	7	7	1 - 2 years	No barriers
				Mexico	4	8	It is already a standard practice	No barriers
				Panama	2	3	> 3 years	Cultural barriers
				Peru	3	7	> 3 years	High costs
				Saint Lucia	7	9	It is already a standard practice	No barriers
				Suriname	3	5	1 - 2 years	Cultural barriers
				Trinidad & Tobago	8	8	It is already a standard practice	No barriers
				Turcs & Caicos	9	9	It is already a standard practice	Cultural barriers
				Uruguay	1	4	> 3 years	High costs
				Venezuela	7	9	< 1 year	No barriers

4	Diagnosis	Include	BP thresholds to consider HTN in the step A (BP 140/90 in general population and SBP 130 in high CVD risk).	Anguilla	9	9	It is already a standard practice	No barriers
				Argentina	2	9	1 - 2 years	Cultural barriers
				Bahamas	9	9	It is already a standard practice	No barriers
				Barbados	8	9	It is already a standard practice	No barriers
				Belize	6	9	It is already a standard practice	No barriers
				Bermuda	5	3	> 3 years	Cultural barriers
				Bolivia	2	7	1 - 2 years	Regulatory barriers
				Brazil	9	9	It is already a standard practice	No barriers
				BVI	5	8	1 - 2 years	Cultural barriers
				Chile	9	9	1 - 2 years	Regulatory barriers
				Cuba	9	9	It is already a standard practice	No barriers
				Dominican Republic	9	9	It is already a standard practice	No barriers
				Ecuador	8	8	It is already a standard practice	No barriers
				El Salvador	9	9	It is already a standard practice	No barriers
				Grenada	9	9	It is already a standard practice	No barriers
				Guatemala	9	9	It is already a standard practice	No barriers
				Guyana	9	7	1 - 2 years	No barriers
				Mexico	9	9	It is already a standard practice	No barriers
				Panama	5	3	> 3 years	Regulatory barriers
				Peru	9	9	It is already a standard practice	No barriers
				Saint Lucia	7	9	It is already a standard practice	No barriers
				Suriname	9	9	It is already a standard practice	No barriers
				Trinidad & Tobago	8	8	It is already a standard practice	No barriers
				Turcs & Caicos	9	9	It is already a standard practice	No barriers
				Uruguay	4	6	> 3 years	Cultural barriers
				Venezuela	8	9	< 1 year	No barriers
5		Modify		Anguilla	8	8	It is already a standard practice	No barriers

	**Risk assessment**		**Clarify the CKD definition as follows: eGFR < 60 ml/min and/or AlbU/CrU index ≥ 30 mg/g.**	Argentina	4	9	< 1 year	High costs
				Bahamas	9	9	It is already a standard practice	No barriers
				Barbados	5	8	< 1 year	No barriers
				Belize	3	9	< 1 year	No barriers
				Bermuda	2	5	> 3 years	Cultural barriers
				Bolivia	1	7	> 3 years	High costs
				Brazil	3	9	1 - 2 years	Regulatory barriers
				BVI	3	7	1 - 2 years	Cultural barriers
				Chile	9	7	It is already a standard practice	Cultural barriers
				Cuba	7	9	It is already a standard practice	No barriers
				Dominican Republic	3	9	1 - 2 years	No barriers
				Ecuador	8	8	It is already a standard practice	High costs
				El Salvador	9	9	It is already a standard practice	No barriers
				Grenada	9	9	It is already a standard practice	No barriers
				Guatemala	5	9	> 3 years	Cultural barriers
				Guyana	9	7	1 - 2 years	No barriers
				Mexico	1	8	< 1 year	No barriers
				Panama	9	9	It is already a standard practice	No barriers
				Peru	5	9	1 - 2 years	No barriers
				Saint Lucia	7	9	It is already a standard practice	No barriers
				Suriname	1	5	1 - 2 years	Cultural barriers
				Trinidad & Tobago	8	8	It is already a standard practice	No barriers
				Turcs & Caicos	9	9	It is already a standard practice	No barriers
				Uruguay	1	4	> 3 years	Cultural barriers
				Venezuela	8	9	< 1 year	No barriers
6	**Risk assessment**	**Modify**	**BP goals in elderly patients to SBP <130**	Anguilla	3	3	< 1 year	Regulatory barriers
				Argentina	2	4	> 3 years	Cultural barriers

			**(age ≥ 65 years as a high CVD-risk equivalent).**	Bahamas	9	9	It is already a standard practice	No barriers
				Barbados	3	8	< 1 year	No barriers
				Belize	2	9	< 1 year	No barriers
				Bermuda	5	3	> 3 years	Cultural barriers
				Bolivia	4	9	1 - 2 years	No barriers
				Brazil	7	7	> 3 years	No barriers
				BVI	4	8	< 1 year	Cultural barriers
				Chile	1	1	> 3 years	Cultural barriers
				Cuba	7	9	< 1 year	No barriers
				Dominican Republic	7	9	1 - 2 years	No barriers
				Ecuador	5	8	1 - 2 years	Regulatory barriers
				El Salvador	1	8	< 1 year	Regulatory barriers
				Grenada	6	8	1 - 2 years	Cultural barriers
				Guatemala	9	9	It is already a standard practice	No barriers
				Guyana	9	9	It is already a standard practice	No barriers
				Mexico	1	8	< 1 year	No barriers
				Panama	1	1	> 3 years	Regulatory barriers
				Peru	1	9	1 - 2 years	No barriers
				Saint Lucia	5	9	< 1 year	Cultural barriers
				Suriname	5	9	1 - 2 years	Cultural barriers
				Trinidad & Tobago	8	8	It is already a standard practice	No barriers
				Turcs & Caicos	1	9	< 1 year	No barriers
				Uruguay	1	3	> 3 years	Cultural barriers
				Venezuela	7	9	< 1 year	No barriers
**7**	**Risk assessment**	**Modify**	**Clarify the CVD risk approach for young adults (18 - 40 years)**	Anguilla	1	1	1 - 2 years	Regulatory barriers
				Argentina	1	1	> 3 years	Cultural barriers
				Bahamas	8	9	1 - 2 years	No barriers

			who are not covered by the CVD risk charts.	Barbados	2	7	1 - 2 years	High costs
				Belize	1	9	< 1 year	No barriers
				Bermuda	1	5	> 3 years	Cultural barriers
				Bolivia	1	9	< 1 year	No barriers
				Brazil	9	9	It is already a standard practice	No barriers
				BVI	2	8	1 - 2 years	Cultural barriers
				Chile	9	9	It is already a standard practice	No barriers
				Cuba	4	8	< 1 year	No barriers
				Dominican Republic	5	9	1 - 2 years	No barriers
				Ecuador	4	4	> 3 years	Regulatory barriers
				El Salvador	1	8	1 - 2 years	Regulatory barriers
				Grenada	9	9	It is already a standard practice	No barriers
				Guatemala	5	9	1 - 2 years	Cultural barriers
				Guyana	6	6	1 - 2 years	No barriers
				Mexico	1	8	< 1 year	No barriers
				Panama	5	1	> 3 years	Regulatory barriers
				Peru	1	9	1 - 2 years	No barriers
				Saint Lucia	4	7	1 - 2 years	No barriers
				Suriname	4	9	1 - 2 years	Cultural barriers
				Trinidad & Tobago	8	8	It is already a standard practice	No barriers
				Turcs & Caicos	1	9	< 1 year	No barriers
				Uruguay	1	9	< 1 year	No barriers
				Venezuela	6	9	< 1 year	No barriers
8	**Risk assessment**	**Include**	**Screening of CKD by urine albumin-creatinine ratio (uACR) and estimated**	Anguilla	9	9	It is already a standard practice	No barriers
				Argentina	3	6	> 3 years	High costs
				Bahamas	9	9	It is already a standard practice	No barriers
				Barbados	3	8	< 1 year	Cultural barriers

			**Glomerular Filtration Rate (eGFR).**	Belize	**8**	**9**	< 1 year	No barriers
				Bermuda	**1**	**4**	> 3 years	Regulatory barriers
				Bolivia	**1**	**1**	> 3 years	Regulatory barriers
				Brazil	**3**	**8**	> 3 years	Regulatory barriers
				BVI	**8**	**8**	It is already a standard practice	High costs
				Chile	**9**	**9**	It is already a standard practice	No barriers
				Cuba	**4**	**7**	< 1 year	High costs
				Dominican Republic	**4**	**7**	> 3 years	High costs
				Ecuador	**7**	**5**	< 1 year	High costs
				El Salvador	**9**	**8**	It is already a standard practice	High costs
				Grenada	**9**	**9**	It is already a standard practice	No barriers
				Guatemala	**1**	**3**	> 3 years	High costs
				Guyana	**1**	**3**	1 - 2 years	High costs
				Mexico	**5**	**5**	1 - 2 years	High costs
				Panama	**1**	**7**	1 - 2 years	Cultural barriers
				Peru	**3**	**9**	> 3 years	High costs
				Saint Lucia	**5**	**8**	1 - 2 years	High costs
				Suriname	**1**	**5**	1 - 2 years	Cultural barriers
				Trinidad & Tobago	**5**	**1**	> 3 years	High costs
				Turcs & Caicos	**9**	**5**	> 3 years	Regulatory barriers
				Uruguay	**1**	**4**	> 3 years	High costs
				Venezuela	**4**	**9**	< 1 year	No barriers
**9**	**Risk assessment**	**Include**	**A Recommendation to measure HTN-mediated organ damage with ECG in high CVD risk patients.**	Anguilla	**3**	**4**	> 3 years	High costs
				Argentina	**3**	**8**	1 - 2 years	No barriers
				Bahamas	**9**	**9**	It is already a standard practice	No barriers
				Barbados	**5**	**8**	< 1 year	High costs
				Belize	**7**	**9**	1 - 2 years	High costs

				Bermuda	5	2	> 3 years	High costs
				Bolivia	1	1	> 3 years	High costs
				Brazil	7	7	1 - 2 years	No barriers
				BVI	9	9	It is already a standard practice	No barriers
				Chile	9	9	It is already a standard practice	No barriers
				Cuba	7	8	It is already a standard practice	No barriers
				Dominican Republic	5	7	1 - 2 years	High costs
				Ecuador	8	6	> 3 years	High costs
				El Salvador	5	5	1 - 2 years	High costs
				Grenada	6	8	< 1 year	Cultural barriers
				Guatemala	1	2	> 3 years	High costs
				Guyana	3	3	1 - 2 years	High costs
				Mexico	7	8	1 - 2 years	High costs
				Panama	5	7	1 - 2 years	High costs
				Peru	1	5	> 3 years	High costs
				Saint Lucia	8	9	< 1 year	No barriers
				Suriname	1	5	1 - 2 years	High costs
				Trinidad & Tobago	7	7	It is already a standard practice	No barriers
				Turcs & Caicos	8	9	It is already a standard practice	No barriers
				Uruguay	5	9	1 - 2 years	No barriers
				Venezuela	8	9	1 - 2 years	High costs
**10**	**Risk assessment**	**Include**	**Screening for dyslipidemia and diabetes among patients with hypertension and obesity.**	Anguilla	9	9	It is already a standard practice	No barriers
				Argentina	5	8	1 - 2 years	High costs
				Bahamas	7	7	< 1 year	No barriers
				Barbados	8	8	It is already a standard practice	No barriers
				Belize	8	9	It is already a standard practice	No barriers
				Bermuda	8	9	1 - 2 years	No barriers

				Bolivia	1	3	> 3 years	High costs
				Brazil	7	7	1 - 2 years	No barriers
				BVI	8	8	It is already a standard practice	High costs
				Chile	9	9	It is already a standard practice	No barriers
				Cuba	5	8	< 1 year	High costs
				Dominican Republic	8	9	It is already a standard practice	No barriers
				Ecuador	7	5	< 1 year	High costs
				El Salvador	8	7	1 - 2 years	High costs
				Grenada	9	9	It is already a standard practice	No barriers
				Guatemala	1	3	> 3 years	High costs
				Guyana	8	5	1 - 2 years	High costs
				Mexico	9	9	It is already a standard practice	No barriers
				Panama	8	9	< 1 year	High costs
				Peru	6	9	> 3 years	High costs
				Saint Lucia	7	9	< 1 year	No barriers
				Suriname	5	9	It is already a standard practice	No barriers
				Trinidad & Tobago	8	8	It is already a standard practice	No barriers
				Turcs & Caicos	9	9	It is already a standard practice	No barriers
				Uruguay	6	9	1 - 2 years	No barriers
				Venezuela	8	9	< 1 year	No barriers
**11**	**Risk assessment**	**Include**	**A case finding strategy (opportunistic screening) for Atrial Fibrillation, in high CVD risk patients of any age and in those ≥ 65 years, using a stepwise approach:**	Anguilla	4	5	1 - 2 years	Regulatory barriers
				Argentina	2	7	1 - 2 years	No barriers
				Bahamas	9	9	It is already a standard practice	No barriers
				Barbados	3	8	1 - 2 years	High costs
				Belize	1	8	1 - 2 years	High costs
				Bermuda	5	2	> 3 years	High costs
				Bolivia	1	1	> 3 years	Regulatory barriers

			**radial pulse palpation to all and ECG in those with 1st test possitive.**	Brazil	1	1	> 3 years	No barriers
				BVI	5	8	1 - 2 years	Cultural barriers
				Chile	9	9	It is already a standard practice	No barriers
				Cuba	4	8	< 1 year	No barriers
				Dominican Republic	1	9	1 - 2 years	No barriers
				Ecuador	6	6	1 - 2 years	High costs
				El Salvador	1	8	1 - 2 years	High costs
				Grenada	8	9	< 1 year	No barriers
				Guatemala	1	5	> 3 years	Cultural barriers
				Guyana	2	2	> 3 years	High costs
				Mexico	1	3	> 3 years	High costs
				Panama	1	5	1 - 2 years	Regulatory barriers
				Peru	1	5	> 3 years	High costs
				Saint Lucia	2	6	1 - 2 years	No barriers
				Suriname	1	4	> 3 years	High costs
				Trinidad & Tobago	8	8	It is already a standard practice	No barriers
				Turcs & Caicos	9	9	It is already a standard practice	No barriers
				Uruguay	2	6	> 3 years	Cultural barriers
				Venezuela	4	9	< 1 year	No barriers
**12**	**Risk assessment**	**Include**	**Closely monitor individuals with a history of hypertension during pregnancy.**	Anguilla	6	6	It is already a standard practice	Regulatory barriers
				Argentina	3	6	> 3 years	Cultural barriers
				Bahamas	1	9	1 - 2 years	No barriers
				Barbados	4	9	< 1 year	No barriers
				Belize	4	9	< 1 year	No barriers
				Bermuda	1	5	< 1 year	No barriers
				Bolivia	1	9	1 - 2 years	No barriers
				Brazil	3	3	> 3 years	No barriers

				BVI	2	8	1 - 2 years	Cultural barriers
				Chile	7	7	1 - 2 years	Regulatory barriers
				Cuba	7	9	It is already a standard practice	No barriers
				Dominican Republic	1	9	1 - 2 years	No barriers
				Ecuador	8	8	It is already a standard practice	No barriers
				El Salvador	1	8	< 1 year	Regulatory barriers
				Grenada	9	9	It is already a standard practice	No barriers
				Guatemala	9	9	It is already a standard practice	No barriers
				Guyana	9	9	It is already a standard practice	No barriers
				Mexico	1	8	< 1 year	No barriers
				Panama	1	9	> 3 years	Regulatory barriers
				Peru	1	9	1 - 2 years	No barriers
				Saint Lucia	3	8	1 - 2 years	No barriers
				Suriname	3	9	1 - 2 years	Cultural barriers
				Trinidad & Tobago	8	8	It is already a standard practice	No barriers
				Turcs & Caicos	9	9	It is already a standard practice	No barriers
				Uruguay	6	9	1 - 2 years	No barriers
				Venezuela	8	9	< 1 year	No barriers
**13**	**Risk assessment**	**Include**	**Warning on the treatment of asymptomatic severe HTN to avoid referral to emergency department and acute treatment with short-acting/parenterally agents.**	Anguilla	7	7	< 1 year	Regulatory barriers
				Argentina	1	1	> 3 years	Cultural barriers
				Bahamas	9	9	It is already a standard practice	No barriers
				Barbados	6	9	It is already a standard practice	No barriers
				Belize	1	9	< 1 year	Cultural barriers
				Bermuda	5	3	> 3 years	High costs
				Bolivia	1	9	1 - 2 years	No barriers
				Brazil	2	1	> 3 years	Regulatory barriers
				BVI	5	8	1 - 2 years	Cultural barriers

				Chile	1	8	1 - 2 years	No barriers
				Cuba	7	9	< 1 year	No barriers
				Dominican Republic	1	1	> 3 years	Regulatory barriers
				Ecuador	8	8	It is already a standard practice	Cultural barriers
				El Salvador	7	7	1 - 2 years	Regulatory barriers
				Grenada	5	9	< 1 year	Cultural barriers
				Guatemala	9	9	It is already a standard practice	No barriers
				Guyana	4	4	1 - 2 years	No barriers
				Mexico	1	9	< 1 year	No barriers
				Panama	7	9	1 - 2 years	Cultural barriers
				Peru	1	5	> 3 years	Cultural barriers
				Saint Lucia	7	8	It is already a standard practice	Cultural barriers
				Suriname	4	9	1 - 2 years	Cultural barriers
				Trinidad & Tobago	8	8	It is already a standard practice	No barriers
				Turcs & Caicos	9	9	It is already a standard practice	No barriers
				Uruguay	1	6	> 3 years	Cultural barriers
				Venezuela	7	9	< 1 year	Cultural barriers
**14**	**Non-pharmacologic treatment**	**Include**	**A recommendation on the consumption of low-sodium / high-potassium salt.**	Anguilla	7	7	It is already a standard practice	Regulatory barriers
				Argentina	7	9	< 1 year	No barriers
				Bahamas	9	9	It is already a standard practice	No barriers
				Barbados	9	9	It is already a standard practice	No barriers
				Belize	1	6	1 - 2 years	Cultural barriers
				Bermuda	7	9	It is already a standard practice	No barriers
				Bolivia	3	9	1 - 2 years	Cultural barriers
				Brazil	7	7	It is already a standard practice	No barriers
				BVI	9	9	It is already a standard practice	No barriers
				Chile	1	5	> 3 years	Cultural barriers

				Cuba	8	9	It is already a standard practice	No barriers
				Dominican Republic	1	1	> 3 years	High costs
				Ecuador	9	8	It is already a standard practice	No barriers
				El Salvador	8	8	< 1 year	No barriers
				Grenada	9	9	It is already a standard practice	No barriers
				Guatemala	4	4	> 3 years	High costs
				Guyana	2	4	1 - 2 years	Regulatory barriers
				Mexico	7	9	It is already a standard practice	Cultural barriers
				Panama	8	9	1 - 2 years	Regulatory barriers
				Peru	1	9	> 3 years	Cultural barriers
				Saint Lucia	9	9	< 1 year	No barriers
				Suriname	5	9	< 1 year	Cultural barriers
				Trinidad & Tobago	8	8	It is already a standard practice	No barriers
				Turcs & Caicos	9	9	It is already a standard practice	Cultural barriers
				Uruguay	6	6	> 3 years	High costs
				Venezuela	8	9	< 1 year	No barriers
**15**	**Non-pharmacologic treatment**	**Include**	**A recommendation on isometric exercise.**	Anguilla	7	6	It is already a standard practice	No barriers
				Argentina	5	8	1 - 2 years	Cultural barriers
				Bahamas	7	9	It is already a standard practice	No barriers
				Barbados	6	9	It is already a standard practice	No barriers
				Belize	3	9	< 1 year	No barriers
				Bermuda	1	1	> 3 years	Cultural barriers
				Bolivia	1	9	< 1 year	No barriers
				Brazil	4	4	> 3 years	Regulatory barriers
				BVI	8	8	It is already a standard practice	No barriers
				Chile	1	1	> 3 years	Cultural barriers
				Cuba	7	9	It is already a standard practice	No barriers

				Dominican Republic	3	9	1 - 2 years	No barriers
				Ecuador	8	8	It is already a standard practice	Cultural barriers
				El Salvador	1	8	< 1 year	Regulatory barriers
				Grenada	4	7	1 - 2 years	Cultural barriers
				Guatemala	7	9	1 - 2 years	Regulatory barriers
				Guyana	9	9	It is already a standard practice	No barriers
				Mexico	1	7	1 - 2 years	Cultural barriers
				Panama	7	8	1 - 2 years	Regulatory barriers
				Peru	4	9	> 3 years	Cultural barriers
				Saint Lucia	4	8	< 1 year	Cultural barriers
				Suriname	5	9	< 1 year	Cultural barriers
				Trinidad & Tobago	8	8	It is already a standard practice	No barriers
				Turcs & Caicos	9	9	It is already a standard practice	Cultural barriers
				Uruguay	6	9	1 - 2 years	No barriers
				Venezuela	6	9	< 1 year	No barriers
**16**	**Non-pharmacologic treatment**	**Include**	**Warning against smoking Cannabis.**	Anguilla	6	5	It is already a standard practice	No barriers
				Argentina	1	1	> 3 years	Cultural barriers
				Bahamas	9	9	It is already a standard practice	No barriers
				Barbados	2	8	< 1 year	Cultural barriers
				Belize	6	9	< 1 year	Cultural barriers
				Bermuda	5	7	< 1 year	No barriers
				Bolivia	1	3	> 3 years	Cultural barriers
				Brazil	6	6	> 3 years	Regulatory barriers
				BVI	8	8	It is already a standard practice	No barriers
				Chile	1	8	1 - 2 years	No barriers
				Cuba	8	9	It is already a standard practice	No barriers
				Dominican Republic	1	9	1 - 2 years	No barriers

				Ecuador	4	4	> 3 years	Regulatory barriers
				El Salvador	5	9	It is already a standard practice	No barriers
				Grenada	9	9	It is already a standard practice	No barriers
				Guatemala	2	9	1 - 2 years	Regulatory barriers
				Guyana	9	7	It is already a standard practice	No barriers
				Mexico	1	7	< 1 year	Cultural barriers
				Panama	1	9	1 - 2 years	Regulatory barriers
				Peru	2	9	1 - 2 years	No barriers
				Saint Lucia	6	9	< 1 year	Cultural barriers
				Suriname	5	8	1 - 2 years	Cultural barriers
				Trinidad & Tobago	8	8	It is already a standard practice	No barriers
				Turcs & Caicos	9	9	It is already a standard practice	Cultural barriers
				Uruguay	1	4	> 3 years	Cultural barriers
				Venezuela	9	9	< 1 year	No barriers
**17**	**Non-pharmacologic treatment**	**Include**	**Warning against Electronic Cigarette use / Vaping.**	Anguilla	7	4	It is already a standard practice	No barriers
				Argentina	4	8	1 - 2 years	Cultural barriers
				Bahamas	9	9	It is already a standard practice	No barriers
				Barbados	2	8	< 1 year	No barriers
				Belize	3	9	< 1 year	No barriers
				Bermuda	2	8	> 3 years	Cultural barriers
				Bolivia	1	9	1 - 2 years	No barriers
				Brazil	9	9	It is already a standard practice	No barriers
				BVI	5	8	< 1 year	Cultural barriers
				Chile	1	8	1 - 2 years	No barriers
				Cuba	7	9	It is already a standard practice	No barriers
				Dominican Republic	1	9	1 - 2 years	No barriers
				Ecuador	8	8	It is already a standard practice	Cultural barriers

				El Salvador	5	9	It is already a standard practice	No barriers
				Grenada	9	9	It is already a standard practice	No barriers
				Guatemala	2	9	1 - 2 years	Regulatory barriers
				Guyana	9	7	It is already a standard practice	No barriers
				Mexico	1	9	< 1 year	No barriers
				Panama	1	9	1 - 2 years	Regulatory barriers
				Peru	1	9	1 - 2 years	No barriers
				Saint Lucia	5	9	< 1 year	No barriers
				Suriname	1	5	1 - 2 years	Cultural barriers
				Trinidad & Tobago	8	8	It is already a standard practice	No barriers
				Turcs & Caicos	9	9	It is already a standard practice	No barriers
				Uruguay	4	8	1 - 2 years	No barriers
				Venezuela	9	9	< 1 year	No barriers
**18**	**Non-pharmacologic treatment**	**Include**	**A recommendation to avoid the sedentary lifestyle.**	Anguilla	9	9	It is already a standard practice	No barriers
				Argentina	8	8	It is already a standard practice	No barriers
				Bahamas	9	9	It is already a standard practice	No barriers
				Barbados	7	9	It is already a standard practice	No barriers
				Belize	5	9	< 1 year	No barriers
				Bermuda	8	9	It is already a standard practice	No barriers
				Bolivia	3	9	< 1 year	No barriers
				Brazil	9	9	It is already a standard practice	No barriers
				BVI	8	8	It is already a standard practice	No barriers
				Chile	1	8	1 - 2 years	No barriers
				Cuba	8	9	It is already a standard practice	No barriers
				Dominican Republic	9	9	It is already a standard practice	No barriers
				Ecuador	8	8	It is already a standard practice	Cultural barriers
				El Salvador	8	8	It is already a standard practice	Cultural barriers

				Grenada	9	9	It is already a standard practice	No barriers
				Guatemala	8	9	1 - 2 years	Regulatory barriers
				Guyana	9	9	It is already a standard practice	No barriers
				Mexico	1	9	< 1 year	No barriers
				Panama	9	9	It is already a standard practice	No barriers
				Peru	5	9	It is already a standard practice	No barriers
				Saint Lucia	5	9	< 1 year	Cultural barriers
				Suriname	5	9	1 - 2 years	Cultural barriers
				Trinidad & Tobago	8	8	It is already a standard practice	No barriers
				Turcs & Caicos	9	9	It is already a standard practice	Cultural barriers
				Uruguay	6	9	1 - 2 years	No barriers
				Venezuela	9	9	< 1 year	No barriers
19	**Non-pharmacologic treatment**	**Include**	**A recommendation on exercise prescription.**	Anguilla	7	5	It is already a standard practice	No barriers
				Argentina	8	8	It is already a standard practice	No barriers
				Bahamas	9	9	It is already a standard practice	No barriers
				Barbados	2	7	1 - 2 years	Regulatory barriers
				Belize	5	9	< 1 year	No barriers
				Bermuda	1	3	> 3 years	Cultural barriers
				Bolivia	3	9	< 1 year	No barriers
				Brazil	9	9	It is already a standard practice	No barriers
				BVI	2	8	1 - 2 years	Cultural barriers
				Chile	9	9	It is already a standard practice	No barriers
				Cuba	8	9	It is already a standard practice	No barriers
				Dominican Republic	9	9	It is already a standard practice	No barriers
				Ecuador	8	8	It is already a standard practice	Cultural barriers
				El Salvador	8	8	It is already a standard practice	Cultural barriers
				Grenada	1	7	1 - 2 years	Cultural barriers

				Guatemala	8	9	1 - 2 years	No barriers
				Guyana	9	7	It is already a standard practice	No barriers
				Mexico	1	7	< 1 year	Cultural barriers
				Panama	9	9	It is already a standard practice	No barriers
				Peru	5	9	It is already a standard practice	No barriers
				Saint Lucia	4	9	< 1 year	Cultural barriers
				Suriname	6	9	It is already a standard practice	No barriers
				Trinidad & Tobago	8	8	It is already a standard practice	No barriers
				Turcs & Caicos	1	9	< 1 year	Cultural barriers
				Uruguay	8	9	It is already a standard practice	No barriers
				Venezuela	9	9	< 1 year	No barriers
**20**	**Pharmacologic treatment**	**Reinforce**	**Use of single-pill combination for hypertension treatment.**	Anguilla	3	3	1 - 2 years	Regulatory barriers
				Argentina	2	7	> 3 years	Regulatory barriers
				Bahamas	1	8	1 - 2 years	Cultural barriers
				Barbados	6	8	1 - 2 years	High costs
				Belize	2	9	< 1 year	No barriers
				Bermuda	1	2	> 3 years	High costs
				Bolivia	1	7	1 - 2 years	Regulatory barriers
				Brazil	3	3	> 3 years	High costs
				BVI	1	6	< 1 year	Regulatory barriers
				Chile	1	1	> 3 years	High costs
				Cuba	5	7	1 - 2 years	High costs
				Dominican Republic	3	7	1 - 2 years	High costs
				Ecuador	3	4	> 3 years	High costs
				El Salvador	7	7	1 - 2 years	High costs
				Grenada	1	8	< 1 year	No barriers
				Guatemala	1	5	> 3 years	High costs

				Guyana	1	1	> 3 years	High costs
				Mexico	5	8	1 - 2 years	Regulatory barriers
				Panama	1	3	> 3 years	Regulatory barriers
				Peru	1	5	> 3 years	Regulatory barriers
				Saint Lucia	7	9	< 1 year	No barriers
				Suriname	1	2	> 3 years	High costs
				Trinidad & Tobago	1	1	> 3 years	High costs
				Turcs & Caicos	1	6	1 - 2 years	High costs
				Uruguay	1	1	> 3 years	Regulatory barriers
				Venezuela	8	8	< 1 year	No barriers
**21**	**Pharmacologic treatment**	**Modify**	**Add the third drug, at half maximum dose, in the second step of the treatment protocol instead increasing the first two drugs to maximum doses.**	Anguilla	2	4	1 - 2 years	Cultural barriers
				Argentina	1	3	> 3 years	Cultural barriers
				Bahamas	1	7	1 - 2 years	Cultural barriers
				Barbados	2	7	1 - 2 years	Cultural barriers
				Belize	1	9	< 1 year	No barriers
				Bermuda	3	9	< 1 year	No barriers
				Bolivia	1	3	> 3 years	Regulatory barriers
				Brazil	6	6	< 1 year	No barriers
				BVI	1	2	> 3 years	Cultural barriers
				Chile	1	1	> 3 years	No barriers
				Cuba	4	7	< 1 year	No barriers
				Dominican Republic	1	7	1 - 2 years	No barriers
				Ecuador	8	8	It is already a standard practice	High costs
				El Salvador	1	1	> 3 years	Regulatory barriers
				Grenada	7	7	1 - 2 years	High costs
				Guatemala	1	1	> 3 years	Cultural barriers
				Guyana	9	7	It is already a standard practice	No barriers

				Mexico	5	5	It is already a standard practice	Cultural barriers
				Panama	1	5	> 3 years	Regulatory barriers
				Peru	1	9	> 3 years	Cultural barriers
				Saint Lucia	4	7	1 - 2 years	Cultural barriers
				Suriname	1	5	It is already a standard practice	Cultural barriers
				Trinidad & Tobago	8	8	< 1 year	No barriers
				Turcs & Caicos	1	9	< 1 year	No barriers
				Uruguay	1	5	> 3 years	Cultural barriers
				Venezuela	7	9	1 - 2 years	No barriers
**22**	**Pharmacologic treatment**	**Modify**	**Statin dose in secondary prevention to Atorvastatin 80 mg or Rosuvastatin 40 mg.**	Anguilla	2	4	1 - 2 years	Cultural barriers
				Argentina	2	7	1 - 2 years	Cultural barriers
				Bahamas	9	9	It is already a standard practice	No barriers
				Barbados	3	7	1 - 2 years	Cultural barriers
				Belize	1	9	< 1 year	No barriers
				Bermuda	2	9	1 - 2 years	No barriers
				Bolivia	1	5	1 - 2 years	Regulatory barriers
				Brazil	9	9	It is already a standard practice	No barriers
				BVI	9	9	It is already a standard practice	No barriers
				Chile	1	2	> 3 years	High costs
				Cuba	4	6	1 - 2 years	High costs
				Dominican Republic	3	9	1 - 2 years	No barriers
				Ecuador	8	8	It is already a standard practice	High costs
				El Salvador	1	1	> 3 years	High costs
				Grenada	5	5	> 3 years	Regulatory barriers
				Guatemala	3	5	> 3 years	High costs
				Guyana	1	1	> 3 years	Regulatory barriers
				Mexico	6	8	< 1 year	Cultural barriers

				Panama	1	9	1 - 2 years	Regulatory barriers
				Peru	1	7	> 3 years	High costs
				Saint Lucia	4	8	1 - 2 years	Cultural barriers
				Suriname	3	7	< 1 year	High costs
				Trinidad & Tobago	6	8	It is already a standard practice	No barriers
				Turcs & Caicos	9	9	It is already a standard practice	No barriers
				Uruguay	3	6	> 3 years	Cultural barriers
				Venezuela	3	9	1 - 2 years	Cultural barriers
23	**Pharmacologic treatment**	**Modify**	**Statin dose in primary prevention to Atorvastatin 40 mg or Rosuvastatin 20 mg.**	Anguilla	2	4	1 - 2 years	Cultural barriers
				Argentina	2	6	< 1 year	High costs
				Bahamas	9	9	It is already a standard practice	No barriers
				Barbados	6	9	< 1 year	Cultural barriers
				Belize	1	9	< 1 year	No barriers
				Bermuda	1	2	> 3 years	Cultural barriers
				Bolivia	1	5	1 - 2 years	Regulatory barriers
				Brazil	7	7	1 - 2 years	High costs
				BVI	9	9	It is already a standard practice	No barriers
				Chile	1	4	> 3 years	High costs
				Cuba	4	6	1 - 2 years	High costs
				Dominican Republic	3	9	1 - 2 years	No barriers
				Ecuador	8	8	It is already a standard practice	High costs
				El Salvador	1	1	> 3 years	High costs
				Grenada	8	8	1 - 2 years	No barriers
				Guatemala	2	6	> 3 years	High costs
				Guyana	9	8	It is already a standard practice	No barriers
				Mexico	9	9	It is already a standard practice	No barriers
				Panama	9	9	It is already a standard practice	High costs

				Peru	2	9	> 3 years	High costs
				Saint Lucia	7	9	< 1 year	No barriers
				Suriname	4	9	1 - 2 years	Cultural barriers
				Trinidad & Tobago	1	1	> 3 years	High costs
				Turcs & Caicos	9	9	It is already a standard practice	No barriers
				Uruguay	6	8	> 3 years	Cultural barriers
				Venezuela	3	9	1 - 2 years	Cultural barriers
**24**	**Pharmacologic treatment**	**Modify**	**Replace current medications in the treatment protocol with polypills (antihypertensive + statin +/- aspirin) for primary and secondary prevention.**	Anguilla	2	4	1 - 2 years	Cultural barriers
				Argentina	1	7	> 3 years	Regulatory barriers
				Bahamas	9	9	It is already a standard practice	No barriers
				Barbados	1	5	> 3 years	High costs
				Belize	1	9	< 1 year	No barriers
				Bermuda	1	1	> 3 years	High costs
				Bolivia	1	5	> 3 years	Regulatory barriers
				Brazil	3	3	> 3 years	High costs
				BVI	1	2	> 3 years	High costs
				Chile	1	1	> 3 years	High costs
				Cuba	2	6	> 3 years	High costs
				Dominican Republic	1	1	> 3 years	Regulatory barriers
				Ecuador	8	8	It is already a standard practice	High costs
				El Salvador	1	1	> 3 years	High costs
				Grenada	1	1	> 3 years	High costs
				Guatemala	1	1	> 3 years	High costs
				Guyana	1	1	It is already a standard practice	No barriers
				Mexico	1	7	1 - 2 years	Regulatory barriers
				Panama	1	1	> 3 years	Regulatory barriers
				Peru	1	5	> 3 years	High costs

				Saint Lucia	2	5	1 - 2 years	High costs
				Suriname	7	9	It is already a standard practice	No barriers
				Trinidad & Tobago	8	8	It is already a standard practice	No barriers
				Turcs & Caicos	1	7	< 1 year	Cultural barriers
				Uruguay	1	1	> 3 years	Regulatory barriers
				Venezuela	1	9	1 - 2 years	No barriers
25	Pharmacologic treatment	Modify	Reduce the intervals between steps for medication intensification to 2 weeks instead of 1 month.	Anguilla	2	3	< 1 year	Cultural barriers
				Argentina	3	6	< 1 year	No barriers
				Bahamas	1	7	< 1 year	Regulatory barriers
				Barbados	4	7	< 1 year	High costs
				Belize	1	9	< 1 year	No barriers
				Bermuda	2	2	> 3 years	High costs
				Bolivia	1	9	< 1 year	No barriers
				Brazil	6	6	> 3 years	High costs
				BVI	2	5	1 - 2 years	Cultural barriers
				Chile	1	2	> 3 years	High costs
				Cuba	6	8	< 1 year	No barriers
				Dominican Republic	1	9	1 - 2 years	No barriers
				Ecuador	7	5	1 - 2 years	High costs
				El Salvador	1	4	1 - 2 years	High costs
				Grenada	1	9	< 1 year	No barriers
				Guatemala	1	8	< 1 year	Cultural barriers
				Guyana	1	1	> 3 years	Regulatory barriers
				Mexico	1	5	1 - 2 years	Regulatory barriers
				Panama	9	9	It is already a standard practice	No barriers
				Peru	1	9	> 3 years	Cultural barriers
				Saint Lucia	2	6	1 - 2 years	Cultural barriers

				Suriname	9	9	It is already a standard practice	Cultural barriers
				Trinidad & Tobago	8	8	< 1 year	No barriers
				Turcs & Caicos	9	9	It is already a standard practice	No barriers
				Uruguay	1	3	> 3 years	High costs
				Venezuela	3	8	< 1 year	No barriers
**26**	**Pharmacologic treatment**	**Modify**	**Change the warning "WOMEN of CHILDBEARING AGE" to "WOMEN of CHILDBEARING POTENTIAL"**	Anguilla	3	3	> 3 years	Regulatory barriers
				Argentina	1	8	< 1 year	Cultural barriers
				Bahamas	1	7	1 - 2 years	Regulatory barriers
				Barbados	6	9	< 1 year	No barriers
				Belize	1	9	< 1 year	No barriers
				Bermuda	1	2	> 3 years	Cultural barriers
				Bolivia	1	9	< 1 year	No barriers
				Brazil	7	7	1 - 2 years	No barriers
				BVI	8	8	< 1 year	No barriers
				Chile	1	9	1 - 2 years	No barriers
				Cuba	6	9	< 1 year	No barriers
				Dominican Republic	1	7	1 - 2 years	Cultural barriers
				Ecuador	4	8	< 1 year	No barriers
				El Salvador	9	9	It is already a standard practice	No barriers
				Grenada	9	9	< 1 year	No barriers
				Guatemala	1	8	< 1 year	Cultural barriers
				Guyana	5	5	1 - 2 years	No barriers
				Mexico	1	8	1 - 2 years	Cultural barriers
				Panama	1	1	> 3 years	Regulatory barriers
				Peru	1	9	1 - 2 years	No barriers
				Saint Lucia	4	9	< 1 year	No barriers
				Suriname	4	9	< 1 year	No barriers

				Trinidad & Tobago	8	8	< 1 year	No barriers
				Turcs & Caicos	1	9	> 3 years	Cultural barriers
				Uruguay	1	8	< 1 year	Cultural barriers
				Venezuela	2	9	< 1 year	No barriers
**27**	**Pharmacologic treatment**	**Include**	**Recommendation of Triple FDC for those patients who don´t reach BP control using Double FDC.**	Anguilla	2	4	1 - 2 years	Cultural barriers
				Argentina	1	3	> 3 years	Cultural barriers
				Bahamas	1	7	1 - 2 years	Cultural barriers
				Barbados	1	6	1 - 2 years	High costs
				Belize	2	9	< 1 year	No barriers
				Bermuda	1	1	> 3 years	High costs
				Bolivia	1	1	> 3 years	High costs
				Brazil	3	3	> 3 years	High costs
				BVI	1	3	> 3 years	Regulatory barriers
				Chile	1	1	> 3 years	High costs
				Cuba	5	7	1 - 2 years	High costs
				Dominican Republic	3	7	1 - 2 years	High costs
				Ecuador	8	8	It is already a standard practice	High costs
				El Salvador	2	3	> 3 years	High costs
				Grenada	1	1	> 3 years	High costs
				Guatemala	1	1	> 3 years	High costs
				Guyana	9	1	It is already a standard practice	No barriers
				Mexico	8	9	It is already a standard practice	No barriers
				Panama	1	1	> 3 years	Regulatory barriers
				Peru	1	5	> 3 years	Regulatory barriers
				Saint Lucia	4	6	1 - 2 years	High costs
				Suriname	1	2	> 3 years	High costs
				Trinidad & Tobago	1	1	> 3 years	High costs

				Turcs & Caicos	9	9	It is already a standard practice	No barriers
				Uruguay	1	1	> 3 years	Regulatory barriers
				Venezuela	7	9	1 - 2 years	No barriers
**28**	**Pharmacologic treatment**	**Include**	**Spironolactone in patients with 3 drugs at maximum doses and lack of HTN control.**	Anguilla	2	4	1 - 2 years	Cultural barriers
				Argentina	1	3	> 3 years	Cultural barriers
				Bahamas	7	9	It is already a standard practice	No barriers
				Barbados	2	6	1 - 2 years	Cultural barriers
				Belize	1	9	< 1 year	No barriers
				Bermuda	7	8	1 - 2 years	No barriers
				Bolivia	1	1	> 3 years	Regulatory barriers
				Brazil	7	7	< 1 year	No barriers
				BVI	1	1	> 3 years	Cultural barriers
				Chile	1	6	> 3 years	Cultural barriers
				Cuba	4	6	< 1 year	No barriers
				Dominican Republic	1	1	> 3 years	Regulatory barriers
				Ecuador	8	8	It is already a standard practice	High costs
				El Salvador	1	1	> 3 years	High costs
				Grenada	9	7	1 - 2 years	No barriers
				Guatemala	1	5	> 3 years	No barriers
				Guyana	1	1	> 3 years	Regulatory barriers
				Mexico	1	5	1 - 2 years	No barriers
				Panama	9	9	It is already a standard practice	No barriers
				Peru	1	9	> 3 years	Cultural barriers
				Saint Lucia	4	8	1 - 2 years	High costs
				Suriname	1	6	< 1 year	Cultural barriers
				Trinidad & Tobago	8	8	< 1 year	High costs
				Turcs & Caicos	1	9	< 1 year	No barriers

				Uruguay	1	8	> 3 years	Cultural barriers
				Venezuela	7	9	1 - 2 years	No barriers
29	**Pharmacologic treatment**	**Include**	**A recommendation for tobacco cessation treatment (e.g. bupropion, varenicline, nicotine substitutes)**	Anguilla	2	4	1 - 2 years	Cultural barriers
				Argentina	4	8	1 - 2 years	Cultural barriers
				Bahamas	4	7	1 - 2 years	High costs
				Barbados	1	4	1 - 2 years	High costs
				Belize	1	8	1 - 2 years	High costs
				Bermuda	1	3	> 3 years	Cultural barriers
				Bolivia	1	5	> 3 years	High costs
				Brazil	9	9	It is already a standard practice	No barriers
				BVI	2	5	> 3 years	Regulatory barriers
				Chile	1	5	> 3 years	High costs
				Cuba	2	6	1 - 2 years	High costs
				Dominican Republic	1	9	> 3 years	Regulatory barriers
				Ecuador	7	7	1 - 2 years	Regulatory barriers
				El Salvador	8	8	< 1 year	Cultural barriers
				Grenada	1	1	> 3 years	Regulatory barriers
				Guatemala	1	4	> 3 years	High costs
				Guyana	9	5	It is already a standard practice	High costs
				Mexico	1	4	> 3 years	High costs
				Panama	9	9	It is already a standard practice	Cultural barriers
				Peru	1	3	> 3 years	High costs
				Saint Lucia	2	8	< 1 year	Regulatory barriers
				Suriname	1	3	1 - 2 years	High costs
				Trinidad & Tobago	4	1	> 3 years	High costs
				Turcs & Caicos	9	9	It is already a standard practice	No barriers
				Uruguay	8	8	It is already a standard practice	Cultural barriers

				Venezuela	1	9	1 - 2 years	No barriers
30	**Pharmacologic treatment**	**Include**	**Recommendation to use iSGLT2 in patients with CKD.**	Anguilla	2	4	1 - 2 years	Cultural barriers
				Argentina	1	5	> 3 years	High costs
				Bahamas	1	7	1 - 2 years	High costs
				Barbados	2	6	1 - 2 years	High costs
				Belize	1	9	1 - 2 years	High costs
				Bermuda	2	5	> 3 years	Cultural barriers
				Bolivia	1	5	> 3 years	Regulatory barriers
				Brazil	9	9	It is already a standard practice	No barriers
				BVI	1	5	> 3 years	Regulatory barriers
				Chile	1	1	> 3 years	High costs
				Cuba	2	5	> 3 years	High costs
				Dominican Republic	1	1	> 3 years	Regulatory barriers
				Ecuador	7	7	1 - 2 years	High costs
				El Salvador	7	7	1 - 2 years	High costs
				Grenada	1	1	> 3 years	High costs
				Guatemala	2	6	> 3 years	High costs
				Guyana	9	5	It is already a standard practice	High costs
				Mexico	5	8	1 - 2 years	Regulatory barriers
				Panama	1	7	1 - 2 years	High costs
				Peru	1	5	> 3 years	High costs
				Saint Lucia	2	7	1 - 2 years	High costs
				Suriname	1	2	> 3 years	High costs
				Trinidad & Tobago	6	6	It is already a standard practice	No barriers
				Turcs & Caicos	9	9	It is already a standard practice	No barriers
				Uruguay	1	1	> 3 years	High costs
				Venezuela	1	9	1 - 2 years	No barriers

31	Pharmacologic treatment	Include	Recommendation to use iSGLT2 in patients with heart failure, regardless of ejection fraction.	Anguilla	2	4	1 - 2 years	Cultural barriers
				Argentina	1	1	> 3 years	High costs
				Bahamas	1	7	1 - 2 years	High costs
				Barbados	4	7	< 1 year	High costs
				Belize	1	9	1 - 2 years	High costs
				Bermuda	2	5	> 3 years	Cultural barriers
				Bolivia	1	5	> 3 years	High costs
				Brazil	9	9	It is already a standard practice	No barriers
				BVI	1	5	> 3 years	Regulatory barriers
				Chile	1	3	> 3 years	High costs
				Cuba	2	5	> 3 years	High costs
				Dominican Republic	1	1	> 3 years	Regulatory barriers
				Ecuador	7	7	1 - 2 years	High costs
				El Salvador	1	1	> 3 years	High costs
				Grenada	1	1	> 3 years	High costs
				Guatemala	2	6	> 3 years	High costs
				Guyana	9	5	It is already a standard practice	High costs
				Mexico	1	8	1 - 2 years	Regulatory barriers
				Panama	1	7	1 - 2 years	High costs
				Peru	1	5	> 3 years	High costs
				Saint Lucia	2	7	1 - 2 years	High costs
				Suriname	1	1	> 3 years	High costs
				Trinidad & Tobago	5	5	1 - 2 years	High costs
				Turcs & Caicos	9	9	It is already a standard practice	No barriers
				Uruguay	1	1	> 3 years	High costs
				Venezuela	2	8	1 - 2 years	No barriers
32		Include		Anguilla	2	4	1 - 2 years	Cultural barriers

	Pharmacologic treatment		Recommendation to use iSGLT2 in patients with diabetes and established CVD.	Argentina	1	3	> 3 years	High costs
				Bahamas	1	7	1 - 2 years	High costs
				Barbados	3	6	1 - 2 years	High costs
				Belize	1	9	1 - 2 years	High costs
				Bermuda	2	5	> 3 years	Cultural barriers
				Bolivia	1	4	> 3 years	Regulatory barriers
				Brazil	9	9	It is already a standard practice	No barriers
				BVI	1	5	> 3 years	Regulatory barriers
				Chile	1	1	> 3 years	High costs
				Cuba	2	5	> 3 years	High costs
				Dominican Republic	1	1	> 3 years	High costs
				Ecuador	7	7	1 - 2 years	High costs
				El Salvador	1	5	> 3 years	High costs
				Grenada	1	1	> 3 years	High costs
				Guatemala	2	6	> 3 years	High costs
				Guyana	9	5	It is already a standard practice	High costs
				Mexico	5	8	1 - 2 years	Regulatory barriers
				Panama	1	7	1 - 2 years	High costs
				Peru	1	5	> 3 years	High costs
				Saint Lucia	2	6	1 - 2 years	High costs
				Suriname	1	1	> 3 years	High costs
				Trinidad & Tobago	5	4	> 3 years	High costs
				Turcs & Caicos	9	9	It is already a standard practice	No barriers
				Uruguay	1	1	> 3 years	High costs
				Venezuela	1	8	1 - 2 years	No barriers
33	Continuity of care	Modify		Anguilla	3	5	< 1 year	Regulatory barriers
				Argentina	3	7	1 - 2 years	Cultural barriers

				Bahamas	8	8	It is already a standard practice	No barriers
				Barbados	4	8	< 1 year	No barriers
				Belize	1	9	< 1 year	No barriers
				Bermuda	7	8	It is already a standard practice	No barriers
				Bolivia	1	9	1 - 2 years	No barriers
				Brazil	9	9	It is already a standard practice	No barriers
				BVI	5	7	1 - 2 years	Cultural barriers
				Chile	9	9	It is already a standard practice	No barriers
				Cuba	7	9	< 1 year	No barriers
				Dominican Republic	1	9	1 - 2 years	No barriers
				Ecuador	8	8	1 - 2 years	Regulatory barriers
			**Clarify that intensive BP goals only apply to patients <80 years.**	El Salvador	1	8	< 1 year	Regulatory barriers
				Grenada	9	9	< 1 year	No barriers
				Guatemala	1	5	> 3 years	Cultural barriers
				Guyana	1	1	1 - 2 years	No barriers
				Mexico	1	8	< 1 year	No barriers
				Panama	7	7	1 - 2 years	Regulatory barriers
				Peru	1	9	1 - 2 years	No barriers
				Saint Lucia	6	9	< 1 year	No barriers
				Suriname	4	9	< 1 year	Cultural barriers
				Trinidad & Tobago	8	8	< 1 year	No barriers
				Turcs & Caicos	9	9	It is already a standard practice	No barriers
				Uruguay	1	5	< 1 year	Cultural barriers
				Venezuela	3	9	< 1 year	No barriers
**34**	**Continuity of care**	**Include**	**Recommendation on Home BP measurement for**	Anguilla	9	9	It is already a standard practice	No barriers
				Argentina	6	7	It is already a standard practice	High costs
				Bahamas	9	9	It is already a standard practice	No barriers

			treatment monitoring.	Barbados	6	9	< 1 year	Cultural barriers
				Belize	8	9	It is already a standard practice	No barriers
				Bermuda	7	7	1 - 2 years	No barriers
				Bolivia	1	5	1 - 2 years	Cultural barriers
				Brazil	9	9	It is already a standard practice	No barriers
				BVI	9	9	It is already a standard practice	No barriers
				Chile	4	4	> 3 years	Cultural barriers
				Cuba	3	8	< 1 year	High costs
				Dominican Republic	5	9	1 - 2 years	High costs
				Ecuador	8	8	It is already a standard practice	High costs
				El Salvador	1	5	> 3 years	High costs
				Grenada	9	9	It is already a standard practice	No barriers
				Guatemala	2	5	> 3 years	High costs
				Guyana	3	3	1 - 2 years	Regulatory barriers
				Mexico	1	8	< 1 year	Cultural barriers
				Panama	9	7	1 - 2 years	Regulatory barriers
				Peru	1	5	> 3 years	High costs
				Saint Lucia	5	7	1 - 2 years	High costs
				Suriname	3	5	1 - 2 years	Cultural barriers
				Trinidad & Tobago	4	1	> 3 years	High costs
				Turcs & Caicos	9	9	It is already a standard practice	No barriers
				Uruguay	6	9	1 - 2 years	No barriers
				Venezuela	5	9	1 - 2 years	No barriers
35	Continuity of care	Include	Recommendation of using Telemedicine / mHealth apps to monitor adherence	Anguilla	1	1	> 3 years	Regulatory barriers
				Argentina	2	4	> 3 years	High costs
				Bahamas	8	7	< 1 year	No barriers
				Barbados	1	4	> 3 years	Regulatory barriers

			and compliance with recommendations, and to reduce absenteeism.	Belize	1	8	1 - 2 years	High costs
				Bermuda	7	9	It is already a standard practice	No barriers
				Bolivia	1	1	> 3 years	Cultural barriers
				Brazil	9	9	It is already a standard practice	No barriers
				BVI	3	6	1 - 2 years	High costs
				Chile	3	3	> 3 years	Cultural barriers
				Cuba	2	5	1 - 2 years	High costs
				Dominican Republic	1	5	> 3 years	Regulatory barriers
				Ecuador	7	5	> 3 years	High costs
				El Salvador	1	7	> 3 years	High costs
				Grenada	1	2	> 3 years	High costs
				Guatemala	2	5	> 3 years	High costs
				Guyana	3	3	1 - 2 years	High costs
				Mexico	3	7	1 - 2 years	High costs
				Panama	6	7	> 3 years	High costs
				Peru	3	7	> 3 years	High costs
				Saint Lucia	3	6	1 - 2 years	Cultural barriers
				Suriname	1	5	1 - 2 years	Cultural barriers
				Trinidad & Tobago	2	2	> 3 years	High costs
				Turcs & Caicos	1	9	1 - 2 years	High costs
				Uruguay	1	5	> 3 years	High costs
				Venezuela	3	9	1 - 2 years	High costs
36	Continuity of care	Include	Lipid targets in high CVD risk patients.	Anguilla	6	8	It is already a standard practice	Regulatory barriers
				Argentina	5	8	1 - 2 years	No barriers
				Bahamas	9	9	It is already a standard practice	No barriers
				Barbados	6	9	< 1 year	No barriers
				Belize	6	9	< 1 year	High costs

				Bermuda	2	5	1 - 2 years	Cultural barriers
				Bolivia	1	5	> 3 years	Cultural barriers
				Brazil	9	9	It is already a standard practice	No barriers
				BVI	8	8	It is already a standard practice	No barriers
				Chile	9	9	It is already a standard practice	No barriers
				Cuba	7	8	< 1 year	High costs
				Dominican Republic	8	9	It is already a standard practice	No barriers
				Ecuador	7	5	1 - 2 years	High costs
				El Salvador	8	8	It is already a standard practice	High costs
				Grenada	9	9	It is already a standard practice	No barriers
				Guatemala	4	6	> 3 years	High costs
				Guyana	9	8	1 - 2 years	No barriers
				Mexico	1	9	< 1 year	No barriers
				Panama	1	7	1 - 2 years	High costs
				Peru	3	9	> 3 years	High costs
				Saint Lucia	6	9	< 1 year	No barriers
				Suriname	6	9	< 1 year	Cultural barriers
				Trinidad & Tobago	8	8	It is already a standard practice	No barriers
				Turcs & Caicos	9	9	It is already a standard practice	No barriers
				Uruguay	5	8	1 - 2 years	No barriers
				Venezuela	3	9	1 - 2 years	No barriers
**37**	**Continuity of care**	**Include**	**A target time to achieve BP control.**	Anguilla	8	8	It is already a standard practice	No barriers
				Argentina	1	5	1 - 2 years	No barriers
				Bahamas	9	9	It is already a standard practice	No barriers
				Barbados	5	8	< 1 year	High costs
				Belize	2	9	< 1 year	No barriers
				Bermuda	1	5	> 3 years	Cultural barriers

				Bolivia	1	9	1 - 2 years	No barriers
				Brazil	8	8	It is already a standard practice	No barriers
				BVI	5	7	1 - 2 years	Cultural barriers
				Chile	1	6	1 - 2 years	Regulatory barriers
				Cuba	7	8	< 1 year	No barriers
				Dominican Republic	9	9	It is already a standard practice	No barriers
				Ecuador	7	7	1 - 2 years	Regulatory barriers
				El Salvador	1	8	1 - 2 years	Regulatory barriers
				Grenada	6	9	< 1 year	No barriers
				Guatemala	6	9	> 3 years	Cultural barriers
				Guyana	9	9	It is already a standard practice	No barriers
				Mexico	1	8	< 1 year	No barriers
				Panama	9	9	It is already a standard practice	No barriers
				Peru	1	9	1 - 2 years	No barriers
				Saint Lucia	6	9	< 1 year	Cultural barriers
				Suriname	5	9	< 1 year	No barriers
				Trinidad & Tobago	8	8	< 1 year	No barriers
				Turcs & Caicos	9	9	It is already a standard practice	Regulatory barriers
				Uruguay	1	3	> 3 years	High costs
				Venezuela	5	9	< 1 year	No barriers
38	**Continuity of care**	**Include**	**An advice not to discontinue statin therapy once the control target has been reached.**	Anguilla	8	8	It is already a standard practice	No barriers
				Argentina	4	8	1 - 2 years	Cultural barriers
				Bahamas	9	9	It is already a standard practice	No barriers
				Barbados	7	9	It is already a standard practice	No barriers
				Belize	3	9	< 1 year	No barriers
				Bermuda	7	8	It is already a standard practice	No barriers
				Bolivia	1	9	1 - 2 years	No barriers

				Brazil	6	6	1 - 2 years	No barriers
				BVI	4	6	1 - 2 years	Cultural barriers
				Chile	1	9	1 - 2 years	No barriers
				Cuba	7	8	< 1 year	No barriers
				Dominican Republic	1	9	> 3 years	Cultural barriers
				Ecuador	7	7	1 - 2 years	Regulatory barriers
				El Salvador	1	8	< 1 year	Regulatory barriers
				Grenada	9	9	It is already a standard practice	No barriers
				Guatemala	6	9	> 3 years	Cultural barriers
				Guyana	9	9	It is already a standard practice	No barriers
				Mexico	1	8	< 1 year	No barriers
				Panama	1	9	1 - 2 years	Regulatory barriers
				Peru	1	9	1 - 2 years	No barriers
				Saint Lucia	5	9	< 1 year	Cultural barriers
				Suriname	6	9	< 1 year	Cultural barriers
				Trinidad & Tobago	5	2	> 3 years	Cultural barriers
				Turcs & Caicos	9	9	It is already a standard practice	No barriers
				Uruguay	3	6	1 - 2 years	Cultural barriers
				Venezuela	3	9	< 1 year	No barriers
39	Delivery system	Include	Non-physician workers under supervision must follow patients and titrate medication to improve BP control and reduce CVD and mortality.	Anguilla	3	5	> 3 years	Regulatory barriers
				Argentina	2	4	> 3 years	Regulatory barriers
				Bahamas	1	1	> 3 years	Regulatory barriers
				Barbados	1	4	> 3 years	Regulatory barriers
				Belize	1	6	1 - 2 years	Regulatory barriers
				Bermuda	1	1	> 3 years	Regulatory barriers
				Bolivia	1	3	> 3 years	Regulatory barriers
				Brazil	9	9	It is already a standard practice	No barriers

				BVI	1	4	> 3 years	Regulatory barriers
				Chile	5	5	1 - 2 years	Regulatory barriers
				Cuba	2	7	< 1 year	Regulatory barriers
				Dominican Republic	7	9	It is already a standard practice	No barriers
				Ecuador	8	8	It is already a standard practice	No barriers
				El Salvador	5	6	1 - 2 years	Regulatory barriers
				Grenada	9	9	It is already a standard practice	No barriers
				Guatemala	3	8	> 3 years	Cultural barriers
				Guyana	9	7	It is already a standard practice	No barriers
				Mexico	1	5	1 - 2 years	Regulatory barriers
				Panama	1	1	> 3 years	Regulatory barriers
				Peru	1	3	> 3 years	Regulatory barriers
				Saint Lucia	4	7	1 - 2 years	Regulatory barriers
				Suriname	2	9	1 - 2 years	Cultural barriers
				Trinidad & Tobago	1	1	> 3 years	High costs
				Turcs & Caicos	1	1	> 3 years	Regulatory barriers
				Uruguay	1	1	> 3 years	Cultural barriers
				Venezuela	2	9	< 1 year	No barriers
**40**	**Delivery system**	**Include**	**Non-physician workers must perform HTN screening and CVD risk stratification.**	Anguilla	4	7	1 - 2 years	Cultural barriers
				Argentina	8	9	It is already a standard practice	No barriers
				Bahamas	1	1	> 3 years	Regulatory barriers
				Barbados	2	8	> 3 years	Cultural barriers
				Belize	5	9	< 1 year	No barriers
				Bermuda	7	5	> 3 years	Cultural barriers
				Bolivia	4	6	> 3 years	Regulatory barriers
				Brazil	9	9	It is already a standard practice	No barriers
				BVI	6	8	1 - 2 years	Cultural barriers

				Chile	9	9	It is already a standard practice	No barriers
				Cuba	6	8	< 1 year	No barriers
				Dominican Republic	5	9	> 3 years	High costs
				Ecuador	8	8	It is already a standard practice	No barriers
				El Salvador	1	7	1 - 2 years	Regulatory barriers
				Grenada	9	9	It is already a standard practice	No barriers
				Guatemala	6	9	> 3 years	Cultural barriers
				Guyana	9	8	It is already a standard practice	No barriers
				Mexico	1	8	1 - 2 years	Cultural barriers
				Panama	1	2	> 3 years	Regulatory barriers
				Peru	1	3	> 3 years	Regulatory barriers
				Saint Lucia	6	9	< 1 year	No barriers
				Suriname	2	9	1 - 2 years	Cultural barriers
				Trinidad & Tobago	8	6	1 - 2 years	High costs
				Turcs & Caicos	9	9	It is already a standard practice	No barriers
				Uruguay	1	5	> 3 years	Cultural barriers
				Venezuela	6	9	< 1 year	No barriers
**41**	**Delivery system**	**Include**	**Non-physician workers must provide counseling on healthy life-style and medication adherence.**	Anguilla	7	8	It is already a standard practice	No barriers
				Argentina	9	9	It is already a standard practice	No barriers
				Bahamas	1	1	1 - 2 years	Regulatory barriers
				Barbados	5	9	< 1 year	Cultural barriers
				Belize	7	9	< 1 year	No barriers
				Bermuda	8	9	It is already a standard practice	No barriers
				Bolivia	3	9	> 3 years	No barriers
				Brazil	9	9	It is already a standard practice	No barriers
				BVI	9	9	It is already a standard practice	No barriers
				Chile	9	9	It is already a standard practice	No barriers

				Cuba	7	9	It is already a standard practice	No barriers
				Dominican Republic	8	9	It is already a standard practice	No barriers
				Ecuador	8	9	It is already a standard practice	No barriers
				El Salvador	9	9	It is already a standard practice	No barriers
				Grenada	9	9	It is already a standard practice	No barriers
				Guatemala	9	9	It is already a standard practice	No barriers
				Guyana	9	8	It is already a standard practice	No barriers
				Mexico	1	8	1 - 2 years	Cultural barriers
				Panama	7	9	> 3 years	Cultural barriers
				Peru	5	9	1 - 2 years	Cultural barriers
				Saint Lucia	6	9	< 1 year	Cultural barriers
				Suriname	4	9	1 - 2 years	Cultural barriers
				Trinidad & Tobago	8	8	It is already a standard practice	No barriers
				Turcs & Caicos	9	9	It is already a standard practice	No barriers
				Uruguay	6	8	1 - 2 years	No barriers
				Venezuela	8	9	< 1 year	No barriers
**42**	**Vaccines**	**Modify**	**Influenza vaccination to all patients with HTN, even those at low and moderate CVD risk.**	Anguilla	8	6	1 - 2 years	Regulatory barriers
				Argentina	8	9	< 1 year	No barriers
				Bahamas	9	9	It is already a standard practice	No barriers
				Barbados	2	5	1 - 2 years	High costs
				Belize	7	9	< 1 year	No barriers
				Bermuda	8	8	< 1 year	No barriers
				Bolivia	1	1	> 3 years	High costs
				Brazil	9	9	It is already a standard practice	No barriers
				BVI	9	8	It is already a standard practice	Cultural barriers
				Chile	9	9	It is already a standard practice	No barriers
				Cuba	7	8	It is already a standard practice	No barriers

				Dominican Republic	9	9	It is already a standard practice	No barriers
				Ecuador	8	8	It is already a standard practice	Cultural barriers
				El Salvador	7	7	1 - 2 years	High costs
				Grenada	9	9	It is already a standard practice	No barriers
				Guatemala	3	7	> 3 years	Cultural barriers
				Guyana	1	6	> 3 years	High costs
				Mexico	6	9	It is already a standard practice	No barriers
				Panama	9	9	It is already a standard practice	Cultural barriers
				Peru	9	9	It is already a standard practice	No barriers
				Saint Lucia	8	9	It is already a standard practice	No barriers
				Suriname	1	3	1 - 2 years	Cultural barriers
				Trinidad & Tobago	8	8	It is already a standard practice	No barriers
				Turcs & Caicos	9	9	It is already a standard practice	Cultural barriers
				Uruguay	8	9	< 1 year	No barriers
				Venezuela	2	9	< 1 year	No barriers
**43**	**Vaccines**	**Modify**	**Indication for pneumococcus vaccination should exclude patients in primary prevention <65 years.**	Anguilla	1	3	> 3 years	Regulatory barriers
				Argentina	9	9	It is already a standard practice	No barriers
				Bahamas	9	9	It is already a standard practice	No barriers
				Barbados	2	7	1 - 2 years	High costs
				Belize	2	9	< 1 year	No barriers
				Bermuda	5	3	> 3 years	Regulatory barriers
				Bolivia	1	7	1 - 2 years	Regulatory barriers
				Brazil	9	9	It is already a standard practice	No barriers
				BVI	3	7	1 - 2 years	Regulatory barriers
				Chile	9	9	It is already a standard practice	No barriers
				Cuba	4	7	< 1 year	No barriers
				Dominican Republic	8	9	It is already a standard practice	No barriers

				Ecuador	5	1	> 3 years	Regulatory barriers
				El Salvador	9	9	It is already a standard practice	No barriers
				Grenada	1	9	< 1 year	No barriers
				Guatemala	1	5	> 3 years	Cultural barriers
				Guyana	1	1	1 - 2 years	No barriers
				Mexico	1	8	< 1 year	No barriers
				Panama	1	1	> 3 years	Regulatory barriers
				Peru	9	9	It is already a standard practice	No barriers
				Saint Lucia	7	9	It is already a standard practice	No barriers
				Suriname	2	2	> 3 years	High costs
				Trinidad & Tobago	8	8	It is already a standard practice	No barriers
				Turcs & Caicos	1	9	1 - 2 years	High costs
				Uruguay	1	3	> 3 years	Regulatory barriers
				Venezuela	3	9	< 1 year	No barriers
**44**	**System for monitoring**	**Include**	**A message about the importance of registering clinical variables.**	Anguilla	8	7	< 1 year	Regulatory barriers
				Argentina	5	8	It is already a standard practice	Cultural barriers
				Bahamas	9	9	It is already a standard practice	No barriers
				Barbados	2	6	1 - 2 years	High costs
				Belize	3	9	< 1 year	No barriers
				Bermuda	3	3	> 3 years	Cultural barriers
				Bolivia	1	9	1 - 2 years	No barriers
				Brazil	9	9	It is already a standard practice	No barriers
				BVI	1	7	< 1 year	Regulatory barriers
				Chile	1	6	> 3 years	Cultural barriers
				Cuba	8	9	It is already a standard practice	No barriers
				Dominican Republic	1	1	> 3 years	Regulatory barriers
				Ecuador	8	8	It is already a standard practice	No barriers

				El Salvador	1	9	< 1 year	No barriers
				Grenada	6	7	< 1 year	Cultural barriers
				Guatemala	1	6	> 3 years	Cultural barriers
				Guyana	9	7	It is already a standard practice	High costs
				Mexico	1	8	< 1 year	No barriers
				Panama	6	6	> 3 years	Regulatory barriers
				Peru	1	7	> 3 years	Cultural barriers
				Saint Lucia	5	9	< 1 year	No barriers
				Suriname	5	9	< 1 year	No barriers
				Trinidad & Tobago	8	8	It is already a standard practice	No barriers
				Turcs & Caicos	9	9	It is already a standard practice	No barriers
				Uruguay	1	1	> 3 years	Cultural barriers
				Venezuela	8	9	< 1 year	No barriers
45	**System for monitoring**	**Include**	**A message about the relevance of having a strategy of preformance evaluation with feedback.**	Anguilla	5	5	1 - 2 years	Cultural barriers
				Argentina	3	5	> 3 years	Cultural barriers
				Bahamas	9	9	It is already a standard practice	No barriers
				Barbados	1	6	> 3 years	High costs
				Belize	3	9	< 1 year	No barriers
				Bermuda	2	2	> 3 years	Regulatory barriers
				Bolivia	2	9	< 1 year	No barriers
				Brazil	9	9	It is already a standard practice	No barriers
				BVI	1	7	< 1 year	Regulatory barriers
				Chile	1	9	< 1 year	No barriers
				Cuba	8	9	It is already a standard practice	No barriers
				Dominican Republic	1	1	> 3 years	Regulatory barriers
				Ecuador	4	7	< 1 year	Regulatory barriers
				El Salvador	9	9	It is already a standard practice	No barriers

				Grenada	6	7	1 - 2 years	Cultural barriers
				Guatemala	2	8	> 3 years	Cultural barriers
				Guyana	6	5	1 - 2 years	High costs
				Mexico	1	8	< 1 year	No barriers
				Panama	6	6	> 3 years	Regulatory barriers
				Peru	1	5	> 3 years	Regulatory barriers
				Saint Lucia	5	9	< 1 year	No barriers
				Suriname	5	9	< 1 year	No barriers
				Trinidad & Tobago	7	7	< 1 year	No barriers
				Turcs & Caicos	9	9	It is already a standard practice	No barriers
				Uruguay	1	1	> 3 years	No barriers
				Venezuela	7	9	< 1 year	No barriers
