# Supplementary material for: Readiness to deliver integrated cardiovascular, kidney and metabolic care in primary healthcare: phase II of HEARTS 2.0 in 26 countries in the Americas
Source: BMJ Glob Health. 2026 Jan 14;11(1):e021298. doi: 10.1136/bmjgh-2025-021298 (PMC12815231; doi:10.1136/bmjgh-2025-021298)
Supplement: online supplemental file 3 [file bmjgh-11-1-s003.pdf]

In this Excel file, you will find **45 candidate interventions to improve the HEARTS Clinical Pathway**. All of them are supported by evidence and have been agreed upon by a group of international experts in the prevention of cardio-kidney-metabolic diseases and public health. These interventions were grouped into the main areas covered by the **HEARTS Clinical Pathway** [https://iris.paho.org/handle/10665.2/56271?locale-attribute=en], and classified according to the recommended action: "reinforce" or "modify" existing recommendations, or "include" new ones.

It is recommended to bring together a group of experts, decision-makers, and members of Primary Health Care (PHC) teams implementing HEARTS to carry out this exercise. Please, include all persons involved in this exercise in the "Team members" table.

Using the drop-down menus, score (1 = lowest; 9 = highest) each intervention based on **1) the reported level of availability** and **2) the perceived level of feasibility to be implemented** in the PHC settings. Additionally, **3) select the time needed to make it a standard practice** in your network of PHC centers, and **4) select the main barrier** that may limit the effective implementation of these interventions in your country.

ID	Action	Interventions	Area	Reported level of availability in PHC settings (*)	Perceived level of feasibility to be implemented in PHC settings (**)	Time needed to make it a standard practice in PHC settings (***)	Main barrier to implement it in your country (****)
1	Reinforce	Exclusive use of clinically validated Blood Pressure Measuring Devices to accurate BP measurement	Diagnosis				
2	Reinforce	Recommendations to improve BP measurement (unobserved, quiet room, etc).	Diagnosis				
3	Include	Expand HTN screening in the community served by each PHC (community engagement).	Diagnosis				
4	Include	BP thresholds to consider HTN in the step A (BP 140/90 in general population and SBP 130 in high CVD risk).	Diagnosis				
5	Modify	Clarify the CKD definition as follows: eGFR < 60 ml/min and/or AlbU/CrU index ≥ 30 mg/g.	Risk assessment				
6	Modify	BP goals in elderly patients to SBP <130 (age ≥ 65 years as a high CVD-risk equivalent).	Risk assessment				
7	Modify	Clarify the CVD risk approach for young adults (18 - 40 years) who are not covered by the CVD risk charts.	Risk assessment				
8	Include	Screening of CKD by urine albumin-creatinine ratio (uACR) and estimated Glomerular Filtration Rate (eGFR).	Risk assessment				
9	Include	A Recommendation to measure HTN-mediated organ damage with ECG in high CVD risk patients.	Risk assessment				
10	Include	Screening for dyslipidemia and diabetes among patients with hypertension and obesity.	Risk assessment				
11	Include	A case finding strategy (opportunistic screening) for Atrial Fibrillation, in high CVD risk patients of any age and in those ≥ 65 years, using a stepwise approach: radial pulse palpation to all and ECG in those with 1st test possitive.	Risk assessment				
12	Include	Closely monitor individuals with a history of hypertension during pregnancy.	Risk assessment				
13	Include	Warning on the treatment of asymptomatic severe HTN to avoid referral to emergency department and acute treatment with short-acting/parenterally agents.	Risk assessment				
14	Include	A recommendation on the consumption of low-sodium / high-potassium salt.	Non-pharmacologic treatment				
15	Include	A recommendation on isometric exercise.	Non-pharmacologic treatment				
16	Include	Warning against smoking Cannabis.	Non-pharmacologic treatment				
17	Include	Warning against Electronic Cigarette use / Vaping.	Non-pharmacologic treatment				
18	Include	A recommendation to avoid the sedentary lifestyle.	Non-pharmacologic treatment				
19	Include	A recommendation on exercise prescription.	Non-pharmacologic treatment				
20	Reinforce	Use of fixed-dose combination.	Pharmacologic treatment				
21	Modify	Add the third drug, at half maximum dose, in the second step of the treatment protocol instead increasing the first two drugs to maximum doses.	Pharmacologic treatment				
22	Modify	Statin dose in secondary prevention to Atorvastatin 80 mg or Rosuvastatin 40 mg.	Pharmacologic treatment				
23	Modify	Statin dose in primary prevention to Atorvastatin 40 mg or Rosuvastatin 20 mg.	Pharmacologic treatment				
24	Modify	Replace current medications in the treatment protocol with polypills (antihypertensive + statin +/- aspirin) for primary and secondary prevention.	Pharmacologic treatment				
25	Modify	Reduce the intervals between steps for medication intensification to 2 weeks instead of 1 month.	Pharmacologic treatment				
26	Modify	Change the warning "WOMEN of CHILDBEARING AGE" to "WOMEN of CHILDBEARING POTENTIAL"	Pharmacologic treatment				
27	Include	Recommendation of Triple FDC for those patients who don´t reach BP control using Double FDC.	Pharmacologic treatment				
28	Include	Spironolactone in patients with 3 drugs at maximum doses and lack of HTN control.	Pharmacologic treatment				
29	Include	A recommendation for tobacco cessation treatment (e.g. bupropion, varenicline, nicotine substitutes)	Pharmacologic treatment				
30	Include	Recommendation to use iSGLT2 in patients with CKD.	Pharmacologic treatment				

**31**	**Include**	Recommendation to use iSGLT2 in patients with heart failure, regardless of ejection fraction.	**Pharmacologic treatment**				
**32**	**Include**	Recommendation to use iSGLT2 in patients with diabetes and established CVD.	**Pharmacologic treatment**				
**33**	**Modify**	Clarify that intensive BP goals only apply to patients <80 years.	**Continuity of care**				
**34**	**Include**	Recommendation on Home BP measurement for treatment monitoring.	**Continuity of care**				
**35**	**Include**	Recommendation of using Telemedicine / mHealth apps to monitor adherence and compliance with recommendations, and to reduce absenteeism.	**Continuity of care**				
**36**	**Include**	Lipid targets in high CVD risk patients	**Continuity of care**				
**37**	**Include**	A target time to achieve BP control.	**Continuity of care**				
**38**	**Include**	An advice not to discontinue statin therapy once the control target has been reached.	**Continuity of care**				
**39**	**Include**	Non-physician workers under supervision must follow patients and titrate medication to improve BP control and reduce CVD and mortality.	**Delivery system**				
**40**	**Include**	Non-physician workers must perform HTN screening and CVD risk stratification.	**Delivery system**				
**41**	**Include**	Non-physician workers must provide counseling on healthy life-style and medication adherence.	**Delivery system**				
**42**	**Modify**	Influenza vaccination to all patients with HTN, even those at low and moderate CVD risk.	**Immunizations**				
**43**	**Modify**	Indication for pneumococcus vaccination should exclude patients in primary prevention <65 years.	**Immunizations**				
**44**	**Include**	A message about the importance of registering clinical variables.	**System for monitoring**				
**45**	**Include**	A message about the relevance of having a strategy of preformance evaluation with feedback.	**System for monitoring**				

(*) Availability refers to the presence of essential inputs and resources needed to operationalize each intervention, reflecting what is currently in place and accessible within the health system.

(**) Implementation feasibility measures how effectively each intervention can be executed in practice, considering existing infrastructure, service delivery workflows, institutional capacity, and policy frameworks.

(***) A standard practice is defined as a one widely accepted and routinely used by health professionals as the recommended care method.

(****) Select the main barrier preventing the widespread adoption of each intervention. If it is a standard practice or has high availability levels, please select "no barriers".
